# Distinct gut microbiota and health outcomes in asymptomatic infection, viral nucleic acid test re‐positive, and convalescent COVID‐19 cases

**DOI:** 10.1002/mlf2.12022

**Published:** 2022-06-15

**Authors:** Ruqin Lin, Mingzhong Xiao, Shanshan Cao, Yu Sun, Linhua Zhao, Xiaoxiao Mao, Peng Chen, Xiaolin Tong, Zheyuan Ou, Hui Zhu, Dong Men, Xiaodong Li, Yiqun Deng, Xian‐En Zhang, Jikai Wen

**Affiliations:** ^1^ Guangdong Provincial Key Laboratory of Protein Function and Regulation in Agricultural Organisms, College of Life Sciences South China Agricultural University Guangzhou China; ^2^ Hepatic Disease Institute, Hubei Key Laboratory of Theoretical and Applied Research of Liver and Kidney in Traditional Chinese Medicine Hubei Provincial Hospital of Traditional Chinese Medicine Wuhan China; ^3^ State Key Laboratory of Virology, Wuhan Institute of Virology, Center for Biosafety Mega‐Science Chinese Academy of Sciences Wuhan China; ^4^ National Laboratory of Biomacromolecules, Institute of Biophysics Chinese Academy of Sciences Beijing China; ^5^ Department of Endocrinology Guang'anmen Hospital, China Academy of Chinese Medical Sciences Beijing China; ^6^ Guangdong Laboratory for Lingnan Modern Agriculture South China Agricultural University Guangzhou China; ^7^ Hubei Province Academy of Traditional Chinese Medicine Wuhan China; ^8^ Faculty of Synthetic Biology, Shenzhen Institute of Advanced Technology Chinese Academy of Sciences Shenzhen China; ^9^ University of Chinese Academy of Sciences Beijing China

**Keywords:** COVID‐19, gut microbiota, health outcomes, re‐positive, SARS‐CoV‐2

## Abstract

Gut microbiota composition is suggested to associate with coronavirus disease 2019 (COVID‐19) severity, but the impact of gut microbiota on health outcomes is largely unclear. We recruited 81 individuals from Wuhan, China, including 13 asymptomatic infection cases (Group A), 24 COVID‐19 convalescents with adverse outcomes (Group C), 31 severe acute respiratory syndrome coronavirus 2 (SARS‐CoV‐2) re‐positive cases (Group D), and 13 non‐COVID‐19 healthy controls (Group H). The microbial features of Groups A and D were similar and exhibited higher gut microbial diversity and more abundant short‐chain fatty acid (SCFA)‐producing species than Group C. Group C was enriched with opportunistic pathogens and virulence factors related to adhesion and toxin production. The abundance of SCFA‐producing species was negatively correlated, while *Escherichia coli* was positively correlated with adverse outcomes. All three groups (A, C, and D) were enriched with the mucus‐degrading species *Akkermansia muciniphila*, but decreased with *Bacteroides*‐encoded carbohydrate‐active enzymes. The pathways of vitamin B6 metabolic and folate biosynthesis were decreased, while selenocompound metabolism was increased in the three groups. Specifically, the secondary bile acid (BA) metabolic pathway was enriched in Group A. Antibiotic resistance genes were common among the three groups. Conclusively, the gut microbiota was related to the health outcomes of COVID‐19. Dietary supplementations (SCFAs, BA, selenium, folate, vitamin B6) may be beneficial to COVID‐19 patients.

## INTRODUCTION

The coronavirus disease 2019 (COVID‐19) caused by severe acute respiratory syndrome coronavirus 2 (SARS‐CoV‐2) has spread rapidly worldwide. The symptoms of COVID‐19 are fever, cough, and gastrointestinal symptoms, including nausea, vomiting, and diarrhea^1^. A meta‐analysis of 4243 COVID‐19 patients in 60 studies shows that the prevalence of gastrointestinal symptoms is 17.6%, and the positive rate of virus RNA in stool samples is 48.1%^2^. Live viruses are also isolated from the fecal specimens of COVID‐19 patients^3^, ^4^. SARS‐CoV‐2 recognizes the receptor angiotensin‐converting enzyme 2 (ACE2) for entry and the serine protease TMPRSS2 for S‐protein priming^5^. The ACE2 protein is abundantly expressed in the gastrointestinal epithelium^6^, and several colonic cell types are also permissive to SARS‐CoV‐2 infection^7^. Accordingly, acute SARS‐CoV‐2 infection induces gastrointestinal inflammation and systemic inflammatory response^8^. Accumulating evidence unveils that SARS‐CoV‐2 infection affects the compositions and diversities of fecal microbiota^9^, ^10^, ^11^, ^12^, and that the gut microbiota richness is associated with the recovery process of COVID‐19^13^, suggesting that gut microbiota may affect the health outcomes of COVID‐19 patients. However, the effects of microbial functional compositions, such as metabolic pathways, virulence, and resistance genes, on the health outcomes of COVID‐19 patients, are still to be characterized.

SARS‐CoV‐2 infection shows complex clinical symptoms and adverse outcomes, ranging from asymptomatic to severe and even fatal. Some discharged COVID‐19 patients have persistent symptoms beyond 3 or 4 weeks from the onset of acute symptoms and are defined as “postacute COVID‐19 syndrome”^14^. The proportion of COVID‐19 convalescent patients with adverse outcomes is 68% at 6 months and 49% at 12 months^15^. COVID‐19 survivors who are severely ill and have impaired pulmonary diffusion capacities during hospitalization are the main targeted population for the intervention of long‐term recovery^16^. A recent study found that the gut microbiome might affect the susceptibility to postacute COVID‐19 syndrome^17^. Also, SARS‐CoV‐2 nucleic acid test re‐positive is a common clinical phenomenon. An epidemiology study reported that 14% of discharged cases reported re‐positive, but none of them was caused by active reinfection^18^. Overall, the factors that affect different clinical outcomes of SARS‐CoV‐2 infection still need investigation.

In this study, we characterized the compositional and functional differences in gut microbiome among asymptomatic, SARS‐CoV‐2 re‐positive and convalescent COVID‐19 cases. These microbiome profiles revealed that the characteristic gut bacteria and microbial metabolic pathways were related to clinical symptoms and outcomes of COVID‐19 patients. Practical applications such as dietary supplements might alleviate the adverse outcomes amongst COVID‐19 patients.

## RESULTS

### Characteristics of the participants

**Figure 1:**
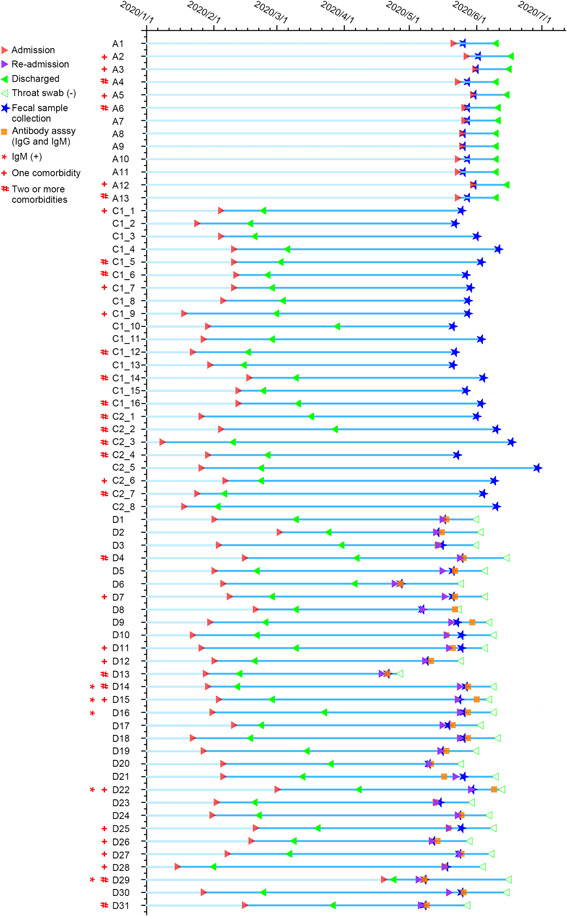
Schematic diagram of fecal sample collection and patient information. Three categories of coronavirus disease 2019 (COVID‐19)‐related cases were recruited, including 13 asymptomatic infection (Group A), 24 COVID‐19 convalescents (Group C), 31 discharged patients with recurrent severe acute respiratory syndrome coronavirus 2 (SARS‐CoV‐2) positivity (Group D). The infection period in Group A was 16–21 days (median 16 days), and the fecal samples were collected within 1–6 days (median 2 days). The acute infection period in Group C was 12–60 days (median 25 days), and the fecal samples were collected after the patients' discharge for 53–128 days (median 92.5 days). The acute infection period in Group D was 5–61 days (median 27 days) and recurred SARS‐CoV‐2 positive on Days 11–106 (median 70) after the first discharge. The fecal samples of Group D were collected on Days 0–7 (median 2) after recurred SARS‐CoV‐2 positive (re‐admission). The re‐positive patients of Group D showed SARS‐CoV‐2 negative on Days 9–42 (median 18) after re‐admission.

We enrolled 81 participants, including 13 asymptomatic infection cases (Group A), 24 convalescent cases with adverse outcomes (Group C), 31 discharged patients with SARS‐CoV‐2 re‐positive (Group D), and 13 non‐COVID‐19 healthy controls (Group H) in this study (Figure S1). The clinical characteristics of participants are shown in Table 1 and the schematic diagram of sample collection is shown in Figure 1. Sex and age were matched among groups. The median ages of Groups A, C, D, and H were 53, 58, 52, and 50 years, and 53.8%, 58.3%, and 45.2% of Group A, C, and D cases had comorbidities, respectively. Hypertension was the most common comorbidity in COVID‐19‐related groups (A, C, and D) and was also matched with Group H. Group A showed no clinical symptoms during the infection period and no sequelae at follow‐up. The count of lymphocytes (LYM) was normal, and only one case (1/13) showed a slightly higher white blood cell (WBC) count in blood at admission (Figure S2A,B). Group C cases had symptoms during hospitalization, including fever (79.2%), respiratory symptoms (66.7%), and gastrointestinal symptoms (50%). Of the cases, 33.3%, 50%, and 16.7% were classified as mild, moderate, and severe COVID‐19 patients at admission, respectively. Group C participants had persistent chest computed tomography (CT) abnormalities (100%) or/and symptoms (79.2%) at the time of fecal sample collection (median discharged time was 92.5 days). Symptoms included respiratory symptoms (41.7%), fatigue (45.8%), muscle soreness (16.7%), sleep disturbances (8.3%), idrosis (8.3%), ageusia (4.2%), and hand paralysis (4.2%). Of the participants, 22.7% showed low levels of LYM or WBC in the blood (Figure S2A,B) and 37.5% had persistent symptoms at the 6‐month follow‐up after being discharged, and the symptoms included fatigue (29.2%), sleep disturbances (8.3%), and ageusia (4.2%). Group D patients that were diagnosed as COVID‐19 cases with moderate symptoms during hospitalization had respiratory symptoms (100%), fever (48.4%) and gastrointestinal symptoms (3.2%) at first admission. These patients were approved to be discharged from the hospital with no obvious clinical symptoms and were later tested negative for SARS‐CoV‐2. However, Group D patients were subsequently re‐positive for SARS‐CoV‐2, and 29% of them had mild clinical symptoms, including breathlessness (19.4%), fatigue (6.5%), and cough (3.2%). Of the patients, 23.3% showed abnormal levels of LYM or WBC in the blood (Figure S2A,B). All Group D patients were positive in the SARS‐CoV‐2 IgG antibody test, and 16.1% of patients were positive in the SARS‐CoV‐2 IgM antibody test in serum at re‐admission. The median SARS‐CoV‐2 re‐positive duration was 18 days. None of them showed clinical symptoms at follow‐up after SARS‐CoV‐2 was negative.

**Table 1:** Clinical information of the study subjects.

Variable	Group A (asymptomatic infection)	Group C (postacute COVID‐19 syndrome)	Group D (SARS‐CoV‐2 test re‐positive)	Group H (non‐COVID‐19 healthy)
Number of cases	13	24	31	13
Male (%)	6 (46.2%)^a^	10 (41.7%)^a^	16 (51.6%)^a^	5 (38.5%)^a^
Age (years)	53 ± 11.5^a^	58 ± 12.5^a^	52 ± 24^a^	50 ± 22.5^a^
Comorbidities (%)	7 (53.8%)^a^	14 (58.3%)^a^	14 (45.2%)^a^	2 (15.4%)^a^
Hypertension	4 (30.8%)^a^	11 (45.8%)^a^	9 (29.0%)^a^	2 (15.4%)^a^
Coronary heart disease	3	0	3	0
Diabetes	1	4	4	0
Fatty liver disease	0	2	1	0
Hyperlipidemia	0	6	0	0
Hyperuricemia	0	3	0	0
Chronic kidney disease	0	1	1	0
Chronic hepatitis B carrier	2	0	0	0
Others	1	1	4	0
Diseases categorized at admission	Asymptomatic infection	Mild: 8 (33.3%); moderate: 12 (50%); severe: 4 (16.7%)	Moderate: 31(100%)	‐
Infection period (days)	16 ± 2^a^	25 ± 14.75^b^	27 ± 22^b^	‐
Symptoms during first hospitalization				
Fever	0	19 (79.2%)	15 (48.4%)	‐
Gastrointestinal symptoms	0	12 (50%)	1 (3.2%)	‐
Respiratory symptoms	0	16 (66.7%)	31 (100%)	‐
Symptoms/chest CT at sample collection				
Chest CT abnormalities	0	22/22 (100%)	8 (25.8%)	‐
Respiratory symptoms	0	10 (41.7%)	9 (22.6%)	‐
Fatigue	0	11 (45.8%)	1 (3.2%)	‐
Muscle soreness	0	4 (16.7%)	0	‐
Sleep disturbances	0	2 (8.3%)	0	‐
Idrosis	0	2 (8.3%)	0	‐
Ageusia	0	1 (4.2%)	0	‐
Hand paralysis	0	1 (4.2%)	0	‐
Symptoms after discharge for 6 months				
Fatigue	0	7 (29.2%)	0	‐
Sleep disturbances	0	2 (8.3%)	0	‐
Ageusia	0	1 (4.2%)	0	‐

Continuous variables are presented as median ± interquartile range. Categorical variables are presented as percentages. Differences between groups were compared by using Kruskal–Wallis for nonnormal continuous variables and the *χ* ^2^ test for categorical variables. Labeled means without a common letter differ, *p* < 0.05.

### Distinct fecal microbial diversity and composition in COVID‐19‐related groups

**Figure 2:**
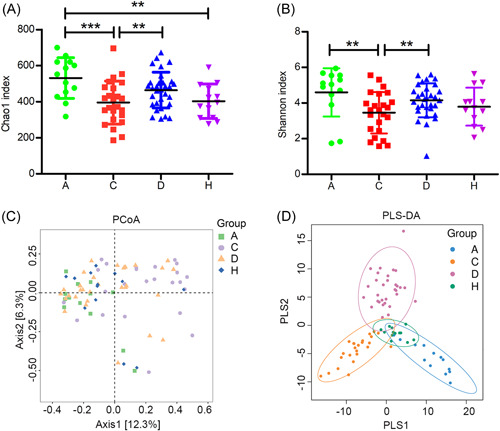
Variation of microbial α‐ and β‐diversity in coronavirus disease 2019 (COVID‐19)‐related and control groups. Microbial α‐diversity was represented by Chao1 index (A) and Shannon index (B) in Group A (*N* = 13), Group C (*N* = 24), Group D (*N* = 31), and Group H (*N* = 13). One‐way analysis of variance with the least significant difference (LSD) test was used to calculate the variation in microbial α‐diversity. Data are shown as mean ± SD. **p < 0.01; ***p < 0.001. Microbial β‐diversity variation was visualized by (C) principal coordinate analysis (PCoA) and (D) partial least‐squares discriminant analysis (PLS‐DA) ordination in four groups. PCoA ordination was calculated based on the Bray–Curtis distance matrix.

**Figure 3:**
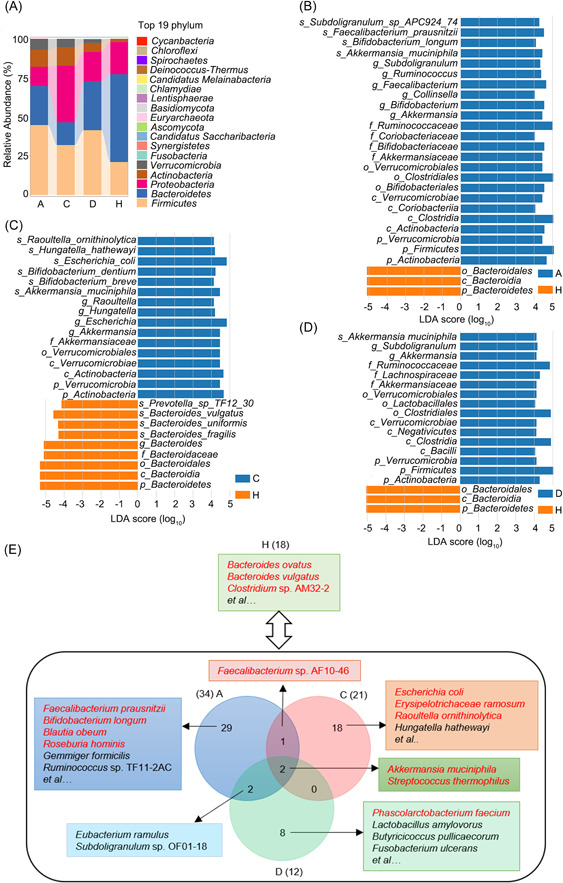
Microbial taxonomic composition and enriched taxa in COVID‐19‐related and control groups. (A) Average relative abundance of microbial phyla in four groups. The Firmicutes/Bacteroidetes ratio was calculated in each group, and a *t* test was performed to compare the differences between the control group (H) and each COVID‐19‐related group (A/C/D). Enriched microbial taxa in Group A (B), Group C (C), and Group D (D) compared with Group H (linear discriminant analysis effect size analysis, least‐squares discriminant analysis (LDA) score > 4, *p* < 0.05). (E) Representative enriched microbial species in Groups A, C, D, and H (LDA score > 3, *p* < 0.05). There were 34, 21, 12, and 18 species enriched in Groups A, C, D, and H, respectively. Two enriched species, *Akkermansia muciniphila* and *Streptococcus thermophilus*, were shared by three COVID‐19‐related groups. Two enriched species, *Eubacterium ramulus* and *Subdoligranulum* sp. OF01‐18, were shared by Groups A and D. The bacterial Growth Rate InDex (GRiD) was calculated based on read coverage differences in bacterial replication origin and terminal regions. A one‐sample *t* test was used to assess the GRiD values. The fast‐growing species are formatted in red font.

A total of 2483 gut microbial species were characterized, including 2434 bacteria, 2 archaea, 14 eukaryotes (13 fungi and 1 oomycete), and 33 DNA viruses. According to the species accumulation curves, the sampling size and sequencing depth were sufficient to represent the overall microbial community (Figure S3A). Group variations in microbial α‐ and β‐diversity were characterized. Groups A and D had a higher Chao1 and Shannon index than Group C (analysis of variance [ANOVA], *p* < 0.05; Figure 2A,B). The principal coordinate analysis (PCoA) plot showed that Groups A and H were mainly located on the left side of axe 1, and Group C was mainly located on the right side of axe 1 (Figure 2C). The partial least‐squares discriminant analysis (PLS‐DA) gave a more apparent separation pattern where Groups A, C, and D had unique microbial compositions that differed from Group H (Figure 2D). Quantitative calculations showed that microbial community differed significantly between Groups A–H, C–H, D–H, A–C, and C–D (permutational multivariate analysis of variance [PERMANOVA] and analysis of similarities [ANOSIM], *p* < 0.05), whereas Group A did not significantly differ from Group D (PERMANOVA, *p* = 0.06; ANOSIM, *p* = 0.30) (Figure 3A). Firmicutes/Bacteroidetes ratio is a widely used marker to assess gut dysbiosis and pathological conditions^19^. Herein, the average Firmicutes/Bacteroidetes ratio for Group H was 0.38 (Figure 3A), similar to the human microbiome project (HMP, 0.30)^20^, whereas the Firmicutes/Bacteroidetes ratios for Groups A, C, and D were 1.80, 2.19, and 1.32, respectively, all higher than Group H (Mann–Whitney test, *p* < 0.05). The abundance of Proteobacteria, a hallmark of gut dysbiosis^21^, was higher in Group C than in other groups (all *p* < 0.05).

Eight hundred and thirty species were common to all four groups, while 74, 207, 242, and 50 species were unique in Groups A, C, D, and H, respectively (Figure S3B). Linear discriminant analysis effect size (LEfSe) analysis was performed to show feature microbial taxa in Groups A, C, and D (Figures 3B–E and Table S1). Overall, *Bacteroides* sp. and *Clostridium* sp. were enriched in Group H, while *Akkermansia muciniphila* and *Streptococcus thermophilus* were enriched in COVID‐19‐related groups (Figure 3E). More specifically, short‐chain fatty acid (SCFA)‐producing and probiotic species were enriched in Group A, including *Faecalibacterium prausnitzii*, *Bifidobacterium longum*, *Blautia obeum*, *Roseburia hominis*, *Gemmiger formicilis*, and *Ruminococcus* sp. TF11‐2AC. Interestingly, a positive correlation was observed between Chao1 index and the abundance of *Faecalibacterium prausnitzii* (*ρ* = 0.579), *Blautia obeum* (*ρ* = 0.723), *Roseburia hominis* (*ρ* = 0.489), and *Gemmiger formicilis* (*ρ* = 0.61), respectively (Spearman's correlation, all *p* < 0.001), suggesting the SCFA‐producing species might promote higher microbial diversity. Other SCFA‐producing gut commensals were also enriched in Group D, including *Phascolrctobacterium faecium*, *Lactobacillus amylovorus*, and *Butyricicoccus pullicaecorum*. The abundance of SCFA‐producing species of these nine species was highest in Group A, followed by Group D and was least in Group C (15.4%, 8.9%, 4.6%, Kruskal–Wallis test, *p* = 0.003) and was negatively correlated with adverse outcomes (logistic regression, odds ratio [OR]: 0.27; 95% confidence interval [CI]: 0.14–0.55; *p *< 0.001). By contrast, opportunistic pathogens were enriched in Group C, including *Escherichia coli*, *Erysipelatoclostridium ramosum*, *Raoultella ornithinolytica*, and *Hungatella hathewayi*. Notably, the abundance of *Escherichia coli* was negatively correlated with Chao1 index (Spearman's correlation, *ρ* = −0.354) and was positively correlated with adverse outcomes (OR: 3.10; 95% CI: 1.43–6.68; *p* = 0.004). Opportunistic pathogen *Fusobacterium ulcerans* was enriched in Group D.

Growth Rate InDex (GRiD) was used to identify the fast‐growing species (Table S2). *Bacteroides ovatus*, *Bacteroides vulgatus*, and *Clostridium* sp. AM32‐2 grew fast in Group H (*t* test, *p* < 0.05). *Faecalibacterium prausnitzii*, *Bifidobacterium longum*, *Blautia obeum*, *Roseburia hominis*, *Faecalibacterium* sp. AF10‐46, and *Streptococcus thermophilus* grew fast in Group A (*p* < 0.05). Similarly, SCFA‐producing species *Phascolarctobacterium faecium* and the shared feature species, *Akkermansia muciniphila* and *Streptococcus thermophilus* grew fast in Group D (*p* < 0.05). Opportunistic pathogens (*Escherichia coli*, *Erysipelotrichaceae ramosum*, and *Raoultella ornithinolytica*) and *A. muciniphila* grew fast in Group C (*p* < 0.05), consistent with the LEfSe and regression analysis. Overall, these results indicated that the SCFA‐producing species were protective factors, while *Escherichia coli* and other opportunistic pathogens were risk factors for adverse outcomes in COVID‐19 patients.

### Characterizing microbial functional pathways in COVID‐19‐related groups

**Figure 4:**
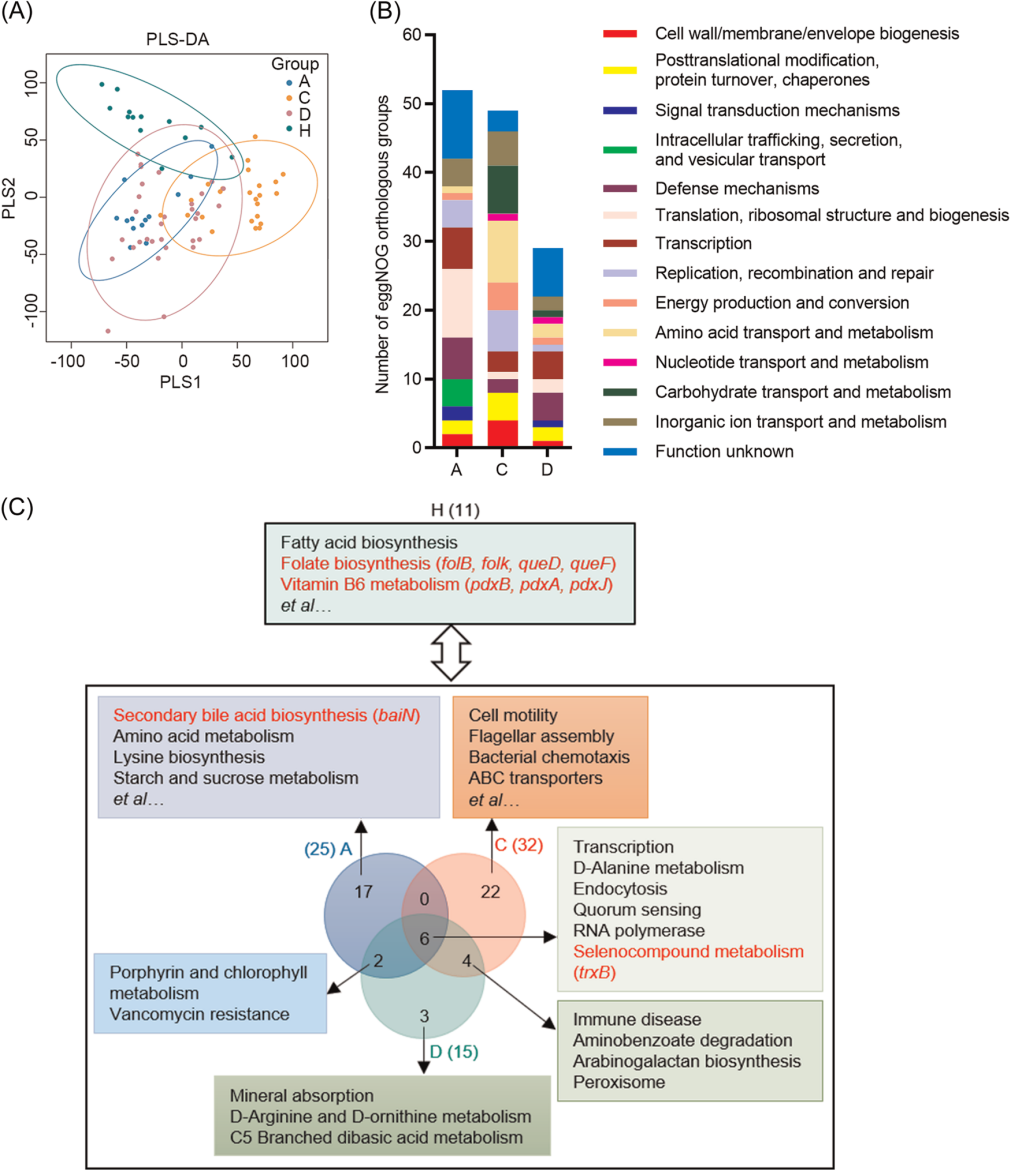
Microbial functional pathways enriched in COVID‐19‐related and control groups, The functional profiling of gut microbiota in COVID‐19‐related and healthy groups based on eggNOG and KO annotation. (A) Partial least‐squares discriminant analysis (PLS‐DA) ordination in Groups A, C, D, and H according to eggNOG annotations. (B) Number of enriched eggNOG orthologous groups in Groups A, C, and D compared with Group H (linear discriminant analysis effect size (LEfSe) analysis, LDA score > 2, *p* < 0.05). There were 53, 49, and 29 enriched eggNOG orthologous groups in Groups A, C, and D, respectively. (C) Enriched microbial functional pathways in Groups A, C, D, and H based on KO annotations (LEfSe analysis, LDA score > 2, *p* < 0.05). There were 25, 32, 15, and 11 enriched functional pathways in Groups A, C, D, and H, respectively. Six enriched pathways were shared by three COVID‐19 groups, two were shared by Groups A and D, and four were shared by Groups C and D. The enriched genes and pathways are formatted in red font.

Next, we profiled the functional pathways in the four groups (Figures S4 and S5). PLS‐DA plot showed that Groups A, C, D, and H were clustered separately (ANOSIM, *p* = 0.001), indicating large compositional variation in functional genes (Figure 4A). According to eggNOG annotation, Groups A and D had more abundant genes involved in defense mechanisms, whereas Group C had more genes associated with amino acid and carbohydrate transport and metabolism and energy production and conversion (LDA score > 2, *p* < 0.05; Figure 4B and Table S3). According to KO annotations, 11 metabolism pathways were enriched in Group H, such as fatty acid biosynthesis, folate biosynthesis, and vitamin B6 metabolism, indicating that these metabolic activities were decreased in COVID‐19‐related groups (LDA score > 2, *p* < 0.05; Table S4). Four genes (*folB*, *folk*, *queD*, and *queF*) were enriched in the folate biosynthesis pathway, and three genes (*pdxB*, *pdxA*, and *pdxJ*) were enriched in the vitamin B6 metabolism pathway in Group H. Compared with Group H, Groups A, C, and D had 25, 32, and 15 characteristic pathways, respectively (Figure 4C). Selenocompound metabolism pathway was enriched in COVID‐19‐related groups and the gene *trxB* was enriched in this pathway. The immune disease pathway was enriched in Groups C and D, indicating potential immune damage by COVID‐19. Nutrient metabolism pathways were enriched in Group A, including secondary bile acid biosynthesis, amino acid metabolism and starch, sucrose metabolism, and lysine biosynthesis. The gene *baiN* was enriched in the secondary bile acid biosynthesis pathway. The mineral absorption pathway was enriched in Group D. Cell motility, flagellar assembly, and bacterial chemotaxis pathways were enriched in Group C, all of which were related to virulence factors, consistent with highly abundant opportunistic pathogens.

### Enrichment of virulence and resistance genes in COVID‐19‐related groups

**Figure 5:**
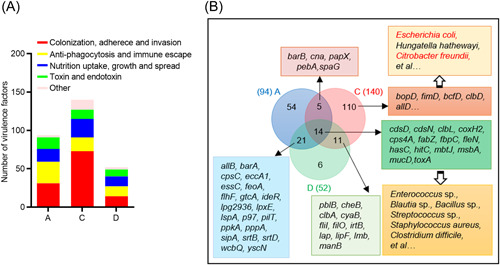
Virulence genes enriched in COVID‐19‐related groups. (A) Number of enriched virulence genes in COVID‐19‐related groups compared with the healthy group (LEfSe analysis, LDA score > 2, *p* < 0.05). The virulent genes were annotated based on the virulence factor database (VFDB). Group A had 31 enriched genes related to colonization, 28 related to anti‐phagocytosis and immune escape, 17 related to nutrition uptake, growth, and spread and 15 related to toxin and endotoxin production. Group C had 73 enriched genes related to colonization, 18 related to anti‐phagocytosis, and immune escape, 24 related to nutrition uptake, growth, and spread, and 12 related to toxin and endotoxin production. Group D had 14 enriched genes related to colonization, 13 related to anti‐phagocytosis and immune escape, 13 related to nutrition uptake, growth, and spread, and 12 related to toxin and endotoxin production. (B) Enriched virulence genes in COVID‐19‐related groups compared with the control group. The COVID‐19‐related groups had 14 shared virulence genes, mainly derived from *Enterococcus* sp., *Blautia* sp., *Bacillus* sp., *Streptococcus* sp., *Staphylococcus aureus*, *Clostridium difficile*, and other opportunistic pathogens, based on sequence similarity analysis. Group C had 110 specific virulence genes, mainly derived from *Escherichia coli*, *Hungatella hathewayi*, and *Citrobacter freundii*.

According to virulence factor database (VFDB) annotations, virulence factors of gut microbiota are divided into five categories: (i) colonization, adhesion, and invasion; (ii) anti‐phagocytosis and immune escape; (iii) nutrition uptake, growth, and spread; (iv) toxin and endotoxin; and (v) others. Compared with Group H, 94, 140, and 52 virulence factors were enriched in Groups A, C, and D, respectively (LDA score > 2, *p* < 0.05; Figure 5A and Table S5). The nutrition uptake genes enriched in three COVID‐19‐related groups were mainly iron intake genes (*fbpC* and *hitC*). More virulence genes were enriched in Group C, specifically colonization, adhesion, and invasion‐associated genes, including *bopD*, *fimD*, and *bcfD*. Blast searching of these virulence sequences indicated that a large proportion of these genes were derived from *Escherichia coli*, *Hungatella hathewayi*, *Citrobacter freundii*, and other opportunistic pathogens (Data not shown). *ecpE*, *fdeC*, and *entE*, genes related to adhesion and enterobactin, were also enriched in Group C and explicitly derived from *Escherichia coli* (LDA score > 2, *p* < 0.05).

The antibiotic resistome of gut microbiota was analyzed by searching against the comprehensive antibiotic resistance database (CARD). Compared with Group H, 67, 66, and 46 genes were enriched in Groups A, C, and D, respectively. There were also 37 resistance genes enriched in Group H. Most of these genes are involved in antibiotic target alteration or antibiotic efflux (LDA score > 2, *p* < 0.05; Figures S6 and S7A), including macrolide, cephalosporin, lincosamide, and carbapenem resistance in COVID‐19‐related groups (Figures S6 and S7B). Overall, COVID‐19‐related groups had more antibiotic target protection genes than Group H. The widespread resistance genes in the gut microbiota of COVID‐19‐related groups indicate that antibiotics should be cautiously used in the course of treatment. Especially for Group C participants, the enriched resistance genes (*otrC*, *oleB*, *msrE*, *salA*, *vmlR*, *vgaE*, *vgaB*, *optrA*, and *poxtA*) were derived from *Escherichia coli*, suggesting that the opportunistic pathogen has the potential to resist antibiotic treatment.

### Carbohydrate‐active enzyme (CAZy) families are altered in COVID‐19‐related groups

**Figure 6:**
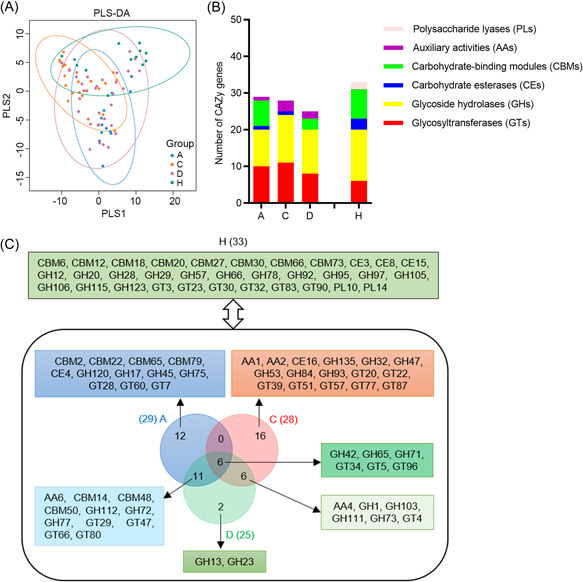
Carbohydrate‐active enzyme (CAZy) families enriched in COVID‐19‐related groups. (A) Partial least‐squares discriminant analysis (PLS‐DA) ordination in Groups A, C, D, and H according to CAZy annotations. (B) Number of enriched CAZy families in COVID‐19‐related groups compared with the control group (LEfSe analysis, LDA score > 2, *p* < 0.05). Thirty‐three CAZy families were enriched in Group H, including 14 glycoside hydrolases (GHs), 8 carbohydrate‐binding modules (CBMs), 6 glycosyltransferases (GTs), 3 carbohydrate esterases (CEs), and 2 polysaccharide lyases (PLs). Twenty‐nine CAZy families were enriched in Group A, including 10 GTs, 10 GHs, 1 CE, 7 CBMs, and 1 auxiliary activities (AAs). Twenty‐eight CAZy enriched in Group C, including 11 GTs, 13 GHs, 1 CE, and 3 AAs. Twenty‐five CAZy enriched in Group D, including 8 GTs, 12 GHs, 3 CBMs, and 2 AAs. (C) Enriched CAZy families in COVID‐19‐related groups compared with the control group. A total of 6 enriched CAZy families were shared by 3 COVID‐19‐related groups, 11 shared by Groups A and D, and 6 shared by Groups C and D.

Human gut microbiota encodes hundreds of CAZy genes to degrade various dietary and carbohydrates, which is important for human health. PLS‐DA and statistical analysis showed overall CAZy variation among groups (Figure 6A, ANOSIM, *p* = 0.001). The relative abundance of glycosyltransferases (GTs), glycoside hydrolases (GHs), carbohydrate‐binding modules (CBMs), carbohydrate esterases (CEs), polysaccharide lyases (PLs), and auxiliary activities (AAs) families are shown in Figure 6B. LEfSe analysis found 33 CAZy families, including 14 GHs, 8 CBMs, 6 GTs, 3 CEs, and 2 PLs were enriched in group H (LDA score > 2, *p* < 0.05; Figure 6C and Table S6), indicating that these CAZy families were decreased in COVID‐19 groups. *Bacteroidetes* encode more CAZy families than other phyla, suggesting that it can use a larger range of carbohydrate substrates. The abundance of CAZy families was decreased in COVID‐19‐related groups, consistent with reduced *Bacteroidetes* in COVID‐19‐related groups. The gut microbiome confers the metabolic abilities to compensate for the paucity of GHs and PLs encoded in the human genome. PL family was not enriched in Groups A, C, and D, suggesting inadequate degradation of polysaccharides by gut microbiota in COVID‐19 patients.

Compared with Group H, 29, 28, and 25 CAZy families were enriched in Groups A, C, and D, respectively (LDA score > 2, *p* < 0.05). GH42, GT34, and GT96 families, enriched in three COVID‐19‐related groups, are related to galactose metabolism. CBMs play a key role in the hydrolysis of cellulose, xylan, and chitin. Seven CBMs families were enriched in Group A, and three of them were common in Group D. CBM14, an enriched CAZy family in Groups A and D, was first identified as an antimicrobial protein with the chitin‐binding ability^22^. There was no characteristic CBM enrichment in Group C, which might lead to poor enzymatic activity in the hydrolysis of cellulose and xylan. GH73 was enriched in Groups C and D, and the enzyme is used during the host‐cell invasion, such as the virulence‐associated peptidoglycan hydrolase^23^, suggesting that the CAZy family might facilitate potential pathogenesis.

## DISCUSSION

The “biodiversity hypothesis” suggests that maintaining microbial diversity (richness) in the gastrointestinal tract is crucial for human health; losing microbial diversity can lead to gut dysbiosis and many chronic, metabolic and immune diseases^24^, ^25^. Two recent studies reported a lower gut microbial diversity in COVID‐19 patients when compared to healthy controls^9^, ^26^, but the pattern was not found in another COVID‐19 gut microbiota survey^27^. In this study, we found that Groups A and D (no symptom after discharge) subjects had a significantly higher microbial diversity than Group C (with adverse outcomes), consistent with that of COVID‐19 patients with lower microbial richness who had worse pulmonary functions^13^. Moreover, Group A had higher α‐diversity than healthy controls. Asymptomatic infections are more common in populations of young and middle‐aged individuals than in the elderly^28^. It has been reported that the viral load of SARS‐CoV‐2 detected in asymptomatic cases was similar to that in symptomatic patients^29^. The asymptomatic and symptomatic cases displayed different gut microbiome, suggesting that gut microbiota may be related to the symptoms or progression of COVID‐19^30^. Thus, the higher microbial richness partially explained the asymptomatic clinical characteristics of participants. COVID‐19 vaccine protects against COVID‐19 by shifting toward more asymptomatic infections^31^. All the cases (including asymptomatic infection cases) enrolled in this study were unvaccinated, indicating the symptom and microbiota variation is not driven by vaccination.

Besides the microbial richness, the β‐diversity indicated the distinct fecal microbial composition in COVID‐19‐related groups. The SCFA‐producing species *Faecalibacterium prausnitzii*, *Blautia obeum*, *Roseburia hominis*, and *Gemmiger formicilis* were enriched in Group A, and their abundance was positively correlated with microbial richness, suggesting that SCFA‐producing species might promote higher microbial diversity and contribute to the positive health consequences. Several other SCFA‐producing species, *Bifidobacterium longum*, *Eubacterium ramulus*, *Pascolarctobacterium faecium*, *Butyricicoccus pullicaecorum*, and *Lactobacillus amylovorus* were also enriched in Groups A and D, and all these nine SCFA‐producing species were negatively correlated with poor prognosis in COVID‐19‐related cases. In contrast, the butyrate‐producing species (including *Faecalibacterium prausnitzii* and *Blautia obeum*) were significantly depleted in patients with postacute COVID‐19 syndrome^17^. SCFAs have antiinflammatory effects by modulating the overproduction of inflammatory cytokines and chemokines during viral infection^32^. The overproduction of inflammatory cytokines, known as “cytokine storm”, can cause tissue damage in COVID‐19 patients and is one of the major reasons for the disease's mortality and morbidity. SCFAs might play an important role in antagonizing the inflammatory response induced by SARS‐CoV‐2. As Groups A and D were asymptomatic or manifested with only mild symptoms despite being tested positive for SARS‐CoV‐2, and since none of them developed sequelae, these results indicate that maintaining balanced and diverse gut microbiota and SCFAs are important in limiting sequelae. In addition, based on the low positive rate of IgM antibody (16.1%) in Group D, and that all of them were home quarantined, the re‐positivity of SARS‐CoV‐2 was unlikely due to reinfection. In a large epidemiologic study, the reinfection incidence in recovered COVID‐19 patients is only 0.02%^33^, consistent with our results. Thus, the persistent viral presence is not associated with adverse health outcomes, suggesting that the gut microbiota is likely a better indicator to predict prognosis than persistent SARS‐CoV‐2 presence. Group C was enriched with opportunistic pathogens, such as *Erysipelatoclostridium ramosum*, *Raoultella ornithinolytica*, *Hungatella hathewayi* and *Escherichia coli*. *Erysipelatoclostridium ramosum* (previously called *Clostridium ramosum*) and *Hungatella hathewayi* (previously called *Clostridium hathewayi*) were the top bacteria that were positively associated with COVID‐19 disease severity^10^. Furthermore, *Erysipelatoclostridium ramosum* was also enriched in patients with postacute COVID‐19 syndrome^17^. These results suggested that *Erysipelatoclostridium ramosum* and *Hungatella hathewayi* were positively associated with postacute COVID‐19 syndrome. *Escherichia coli* was a risk factor for the adverse outcomes in postacute COVID‐19 individuals and was overrepresented with virulence genes related to adhesion and toxins production. The colibactin‐producing *Escherichia coli* strains could cause inflammatory bowel disease (IBD) and colorectal cancer^34^. Indeed, a previous study found that adherent *Escherichia coli* strains in ileal mucosa could cause intestinal inflammatory symptoms, including abdominal pain, diarrhea, and fatigue^35^. Notably, fatigue was the most common symptom in Group C. These results suggest that the opportunistic pathogens *Escherichia coli* may be the risk factor for postacute COVID‐19 syndrome, such as fatigue. According to a previous study^17^ and this study, opportunistic pathogens are the important risk factors in shaping postacute COVID‐19 syndrome. However, it might be inappropriate to use antibiotics in COVID‐19 patients, as the antibiotics resistance genes of gut microbiota were enriched in the COVID‐19‐related cases. Antibiotic therapy might exacerbate gut dysbiosis. A probiotic supplement is one of the possible replacement options for antibiotics.

Compared with the control group, *Bacteroides ovatus* and *Bacteroides vulgatus* were decreased in COVID‐19‐related groups. However, it also reported that the patients with postacute COVID‐19 syndrome had higher levels of *Bacteroides vulgatus* ^17^. This inconsistency might result from the different symptoms of postacute COVID‐19 syndrome and the geographic variation of participants in the two studies. The associated risk factors of the post‐COVID‐19 syndrome may include female sex, more than five early symptoms, early dyspnea, and prior psychiatric disorders^36^. Several studies have demonstrated that *Bacteroides ovatus* and *Bacteroides vulgatus* could reduce intestinal inflammation^37^, ^38^, ^39^. Previous studies reported an inverse correlation of *Bacteroides* with fecal SARS‐CoV‐2 load and ACE2 expression^10^, ^40^. These results suggest that the viral load and entry in COVID‐19 patients may affect *Bacteroides* colonization. *Bacteroidetes* encode many CAZy families, specifically GH and PL, to digest dietary polysaccharides^41^. Low abundant *Bacteroidetes* might limit dietary polysaccharides utilizations in COVID‐19 patients. The role of *Bacteroides*, including *Bacteroides vulgatus* and *Bacteroides ovatus*, in the postacute COVID‐19 syndrome needs to be further clarified. Besides, the mucin‐degrading bacteria, * Akkermansia muciniphila,* were enriched in three COVID‐19‐related groups. It has been reported that virus infection promoted the growth of *Akkermansia muciniphila* in mice^42^. The overrepresentation of *Akkermansia muciniphila* and reduced Firmicute/Bacteroidetes ratio occurred downstream of CD8 T‐cell responses during the early phase of infection with a fast‐spreading and persistent virus^43^. These results indicated that virus infection elicited host immune responses and altered the ecology of the gut microbiota, such as the blooming of *Akkermansia muciniphila*. *Akkermansia muciniphila* degrades the mucus layer to disrupt intestinal barrier function and induces inflammation^44^, and its abundance was positively associated with infection and immune responses in COVID‐19 patients, including expression of interleukin‐1β (IL‐1β) and IL‐6^27^, suggesting adverse effects in COVID‐19 cases. *Akkermansia muciniphila* grew faster in Groups C and D than in Group A, indicating that active *A. muciniphila* may degrade more mucus and induce higher levels of inflammatory cytokines in Groups C and D. SCFAs such as butyrate stimulate mucin production in intestinal epithelial cells, possibly counteracting mucin degradation by *Akkermansia muniniphila* to maintain mucus integrity and microbiota homeostasis, and consequently reducing inflammation in Groups A and D. Targeted quantitative metabolomics showed a drop in SCFAs and changes in several bile acids in the SARS‐CoV‐2‐infected nonhuman primates^45^. The secondary bile acid biosynthesis associated pathways were also enriched in Group A. Secondary bile acids exhibit antiinflammatory effects and restrict alpha virus dissemination by promoting immune response^46^, ^47^. The differences in immune response determine the COVID‐19 disease presentation and severity^48^. It is likely that the enriched SCFAs and secondary bile acid suppress inflammation and improve immunity during SARS‐CoV‐2 infection.

A recent study found that COVID‐19 patients with severe/critical illness showed impaired SCFAs and L‐isoleucine biosynthesis in the gut microbiome^49^. However, a previous study also showed that fecal samples with high SARS‐CoV‐2 infectivity had a higher functional capacity for amino acid biosynthesis^50^. Herein, we found that the amino acid metabolism and lysine biosynthesis associated pathways were enriched in group A. Most amino acids were depleted in the SARS‐CoV‐2‐infected cells, while the aspartate and asparagine were upregulated, likely caused by activation of the cellular integrated stress response^51^. These results suggest that amino acid was essential for the metabolic activity of both SARS‐CoV‐2 and the host cell. Due to the important role of amino acids in maintaining body health, the possible supplementation of amino acids could be considered in COVID‐19 patients. Compared with the control group, vitamin B6 metabolism and folate biosynthesis were decreased in the COVID‐19‐related groups. Vitamin B6 in the body is mainly derived from diet and gut bacteria synthesis via intestinal absorption. Dysregulated gut microbiota and vitamin B6 deficiency led to autism‐like behavior in mice^52^. Vitamin B6 may be useful for COVID‐19 patients in ameliorating the severity of COVID‐19 and its complications^53^. Low levels of serum folate were common among COVID‐19 patients, but were not associated with the clinical outcomes^54^. Folate biosynthesis is a major biochemical feature of the human microbiome, and its deficiency might lead to human health disorders^55^. Furthermore, both folate and vitamin B6 have been demonstrated to have critical roles in supporting the human immune system and reducing the risk of infections^56^. Thus, folate and vitamin B6 supplementations may also help combat COVID‐19. According to the microbial metabolic pathways, the selenocompound metabolism pathway was enriched in COVID‐19‐related groups. A previous study found that 42% of the COVID‐19 patients were selenium deficient^57^. Due to the antioxidant and antiinflammatory properties of selenium, it plays a crucial role in human physiology, such as maintaining the immune system and balance of gut microbiota^58^. Gut microbiota could remove selenium from the host under selenium‐limiting conditions, and dietary selenium could also affect the composition and colonization of gut microbiota^59^. Selenium supplementation should be considered in the dietary supplement of COVID‐19 patients. Overall, increasing gut microbial diversity, either by supplementing with probiotics, prebiotics or dietary supplementations (SCFA, secondary bile acid, selenium, folate, vitamin B6), could be a potential strategy to reduce clinical symptoms and improve prognosis in COVID‐19 patients.

Although many new insights have been proposed, there are several limitations in this study. This study has a small sample size, especially in healthy controls. The Firmicutes/Bacteroidetes ratio for Group H was similar to the ratio described in the HMP project, a large‐scale international collaboration project in characterizing the human microbiome. The ratios for Groups A, C, and D were significantly higher than Group H and HMP reference, indicating the COVID‐19‐related groups deviated from the normal standard. Although the participants were matched for sex, age, and region, gut microbiota can be influenced by other variables, such as host milieu, diet, and lifestyle. Our findings should be further confirmed in the larger cohorts with different populations. Herein, only one fecal sample was collected for each subject, and thus the longitudinal variation of the fecal microbiome cannot be characterized. Moreover, the functional potentials of the gut microbiome were inferred from the shotgun metagenomics. The approach characterizes the relative abundance of the DNA sequences and infers the functional potentials based on genomic data. To validate the abundance analysis results, we applied growth rate analysis to show that most characteristic species were actively replicating, suggesting the active metabolism. Future metabolomics should be a more optimal approach to validate the functional potentials of gut microbial metabolites between COVID‐19 and healthy individuals.

## MATERIALS AND METHODS

### Study design and participants

This study was approved by the Ethics Committee of Hubei Provincial Hospital of Traditional Chinese Medicine (Permit number: HUZY2020‐C21‐01). Participants were recruited from April 17, 2020 to June 30, 2020 in Hubei Provincial Hospital of Traditional Chinese Medicine (Wuhan, China) and were followed up until December 31, 2020. All study participants provided informed consent, and the records were kept at the hospital. The participants were diagnosed, categorized, and received standard treatment according to “diagnosis and treatment guidelines” issued by the National Health Commission of China. We finally enrolled 81 participants, including 13 asymptomatic infections (Group A), 24 convalescent cases with adverse outcomes^14^ (Group C), 31 discharged patients with SARS‐CoV‐2 re‐positive (Group D), and 13 non‐COVID‐19 healthy controls with age‐ and sex‐matched (Group H) (excluding individuals subsequently found to be ineligible for enrollment; Figure S1). Group A participants were tested positive for SARS‐CoV‐2 without clinical symptoms. The fecal/blood samples were collected during the infection period before any drug usage. The fecal/blood samples of Group C were collected in COVID‐19 convalescent cases that were discharged from the hospital and had persisting chest CT abnormalities or symptoms for more than 8 weeks. Group D patients were discharged upon being tested to be negative for SARS‐CoV‐2 and no obvious clinical symptoms, but were later found re‐positive during home quarantine by two consecutive reverse transcription‐polymerase chain reaction tests of throat swabs. The fecal/blood samples were collected when SARS‐CoV‐2 re‐positivity was confirmed. The time of sample collection in participants is shown in Figure 1. No antibiotic was used in the last 3 months before fecal collection. Medical records of patients, including the epidemiological, clinical, laboratory, radiological characteristics, treatment, and outcome data, were reviewed and extracted by experienced clinicians. Clinical outcomes were followed up after patients were discharged for about 6 months by phone. Healthy controls were tested negative for SARS‐CoV‐2 during the COVID‐19 pandemic. The inclusion criteria of non‐COVID‐19 healthy controls are (i) no mental illness, (ii) no underlying infectious or acute disease, and (iii) the residents who lived in the Wuhan area. The exclusion criteria are (i) antibiotic or prebiotic use in the last 3 months, (ii) during pregnancy or lactation, (iii) bowel surgery in the last 6 months and history of gastrointestinal disease, and (iv) known history of severe organ failure.

Blood and fecal samples from patients were collected by hospital staff, and non‐COVID‐19 healthy controls provided fecal samples at home by self‐sampling. Samples were stored at −80°C until processing.

### DNA extraction, library construction, and metagenomic sequencing

Total genomic DNA was extracted from the fecal samples using Stool DNA Kit (Omega Bio‐tek) according to the manufacturer's instructions. The fecal samples were inactivated at 56°C for 30 min before DNA extraction to inactivate SARS‐CoV‐2 viruses. The quantity and quality of the extracted DNA were assessed by Fluorometer (Promega) and agarose gel electrophoresis. For the qualified samples, the shotgun metagenomic libraries were constructed by the TruSeq Nano DNA High Throughput Library Prep Kit (Illumina). Read lengths were 2 × 150 bp with an insert size of ~400 bp. Dual indexed barcodes were applied for sample multiplexing. The prepared libraries were stored in a freezer at −20°C and sequenced by the Illumina HiSeq X Ten platform (Illumina).

### Metagenomic sequencing, assembly, and statistical analysis

Raw reads were demultiplexed according to the barcode information. Adapter sequences were removed by Cutadapt (v1.2.1)^60^. A 5‐bp sliding window algorithm was applied to remove low‐quality regions (average base quality <20) in the raw reads. Trimmed reads with length <50 bp were discarded. Human reads were removed by BMTagger (v.3.101) and KneadData (v.0.9.0). The processed clean reads were deposited in the Genome Sequence Archive (https://bigd.big.ac.cn/gsa) with the accession number CRA003945^61^, ^62^. The clean reads were *de novo* assembled by MEGAHIT (v.1.2.9)^63^. The CDS (coding sequences > 300 bp) regions were predicted by MetaGeneMark (v.3.25)^64^ and clustered by CD‐HIT (v.4.8.1)^65^ to produce a set of nonredundant genes with an amino acid sequence identity cutoff of 90%. The abundance of the nonredundant genes was calculated by coverage function from SOAPdenovo2 (v.1.0)^66^. Microbial taxonomy was annotated by MEGAN (v.6.0)^67^ using a lowest common ancestor algorithm based on searching genes against the NCBI‐NT database (BLASTN, *e *value < 0.001). Microbial functional genes were annotated by searching genes against the KEGG database (release 97.0) and eggNOG (v4.0) by DIAMOND (v.2.0.4) with *e* value < 0.001 and coverage ratio > 40%)^68^, ^69^, ^70^. Microbial nonredundant genes were also searched against CAZy database to describe the structurally related catalytic and carbohydrate‐binding enzymes that degrade, modify, or create glycosidic bonds^71^. Antimicrobial resistance genes were annotated by resistance gene identifier (v.5.2.0) again CARD (v3.1.3)^72^. Microbial virulent factors were annotated by DAMOND (v.2.0.4) against the VFDB (v.2019)^73^.

The characteristic microbial taxa and functional genes/pathways in sample groups were analyzed by LEfSe analysis^74^ on the Galaxy website (http://huttenhower.sph.harvard.edu/galaxy/, v.1.0). Microbial community variation (β‐diversity) was calculated by Bray–Curtis ordination, unconstrained ordination (PCoA), and constrained ordination (PLS‐DA) at the species level among groups^75^, ^76^, ^77^. PERMANOVA/Adonis (10,000 permutations) and ANOSIM were conducted to quantify the microbial taxonomic and functional composition variation between sample groups. The growth rates of characteristic species (LDA > 2 in LEfSe analysis) were calculated by GRiD (v.1.3)^78^. Only species with taxa annotation resolved at the species level were included. Reference genomes were downloaded from the prokaryote database in NCBI. If multiple reference genomes were available, the genome with the smallest scaffold number was chosen. R (v.3.6.1) and Quantitative Insights Into Microbial Ecology (v.1.8.0) were used to analyze and visualize data throughout the study^79^.

Demographic characteristics data were presented as median ± interquartile range and as percentages for categorical variables. Differences between the two groups were compared by using Student's *t* test for normal continuous variables, the Kruskal–Wallis test for nonnormal continuous variables and the *χ* ^2^ test or Fisher's exact test for categorical variables. Binary logistic regression was performed to analyze the associations between the abundance of microbial taxa (log_10_ transformed) and health outcomes. Correlations between enriched species and microbial richness (Chao1 index) were tested with Spearman's correlation. Statistical analyses were performed using SPSS V.21 (SPSS). Differences with a *p* < 0.05 (two‐sided) were considered statistically significant.

## AUTHOR CONTRIBUTIONS

Ruqin Lin, Shanshan Cao, and Yu Sun performed the experiments, data analyses, and drafted the manuscript. Mingzhong Xiao, Linhua Zhao, Peng Chen, Xiaolin Tong, Hui Zhu, and Xiaodong Li recruited subjects and collected clinical samples and data. Xiaoxiao Mao and Zheyuan Ou performed the statistical analysis. Dong Men, Xiaodong Li, Yiqun Deng, Xian‐En Zhang, and Jikai Wen designed and supervised the study. All authors approved the manuscript.

## ETHICS STATEMENT

This study was approved by the Ethics Committee of Hubei Provincial Hospital of Traditional Chinese Medicine (Permit number: HUZY2020‐C21‐01). The study was performed in accordance with the Declaration of Helsinki and the Rules of Good Clinical Practice.

## CONFLICT OF INTERESTS

The authors declare no conflict of interests.

## Supporting information

Supporting information.

## Data Availability

Data are available in a public, open access repository. The processed clean reads were deposited in the Genome Sequence Archive (https://bigd.big.ac.cn/gsa) with the accession number CRA003945.

## References

[1] Guan WJ , Ni ZY , Hu Y , Liang WH , Ou CQ , He JX , et al. Clinical characteristics of coronavirus disease 2019 in China. N Engl J Med. 2020;382:1708–20. 32109013 10.1056/NEJMoa2002032 PMC7092819

[2] Cheung KS , Hung IFN , Chan PPY , Lung KC , Tso E , Liu R , et al. Gastrointestinal manifestations of SARS‐CoV‐2 infection and virus load in fecal samples from a Hong Kong Cohort: systematic review and meta‐analysis. Gastroenterology. 2020;159:81–95. 32251668 10.1053/j.gastro.2020.03.065 PMC7194936

[3] Wang W , Xu Y , Gao R , Lu R , Han K , Wu G , et al. Detection of SARS‐CoV‐2 in different types of clinical specimens. JAMA. 2020;323:1843–4. 32159775 10.1001/jama.2020.3786 PMC7066521

[4] Xiao F , Sun J , Xu Y , Li F , Huang X , Li H , et al. Infectious SARS‐CoV‐2 in feces of patient with severe COVID‐19. Emerging Infect Dis. 2020;26:1920–2. 10.3201/eid2608.200681 PMC7392466 32421494

[5] Hoffmann M , Kleine‐Weber H , Schroeder S , Krüger N , Herrler T , Erichsen S , et al. SARS‐CoV‐2 cell entry depends on ACE2 and TMPRSS2 and is blocked by a clinically proven protease inhibitor. Cell. 2020;181:271–80.e8. 32142651 10.1016/j.cell.2020.02.052 PMC7102627

[6] Xiao F , Tang M , Zheng X , Liu Y , Li X , Shan H . Evidence for gastrointestinal infection of SARS‐CoV‐2. Gastroenterology. 2020;158:1831–3.e3. 32142773 10.1053/j.gastro.2020.02.055 PMC7130181

[7] Han Y , Duan X , Yang L , Nilsson‐Payant BE , Wang P , Duan F , et al. Identification of SARS‐CoV‐2 inhibitors using lung and colonic organoids. Nature. 2021;589:270–5. 33116299 10.1038/s41586-020-2901-9 PMC8034380

[8] Effenberger M , Grabherr F , Mayr L , Schwaerzler J , Nairz M , Seifert M , et al. Faecal calprotectin indicates intestinal inflammation in COVID‐19. Gut. 2020;69:1543–4. 32312790 10.1136/gutjnl-2020-321388 PMC7211078

[9] Ren Z , Wang H , Cui G , Lu H , Wang L , Luo H , et al. Alterations in the human oral and gut microbiomes and lipidomics in COVID‐19. Gut. 2021;70:1253–65. 33789966 10.1136/gutjnl-2020-323826 PMC8042598

[10] Zuo T , Zhang F , Lui GCY , Yeoh YK , Li AYL , Zhan H , et al. Alterations in gut microbiota of patients with COVID‐19 during time of hospitalization. Gastroenterology. 2020;159:944–55.e8. 32442562 10.1053/j.gastro.2020.05.048 PMC7237927

[11] Yeoh YK , Zuo T , Lui GC , Zhang F , Liu Q , Li AY , et al. Gut microbiota composition reflects disease severity and dysfunctional immune responses in patients with COVID‐19. Gut. 2021;70:698–706. 33431578 10.1136/gutjnl-2020-323020 PMC7804842

[12] Zuo T , Wu X , Wen W , Lan P . Gut microbiome alterations in COVID‐19. Genomics Proteomics Bioinformatics . 2021;S1672–0229:00206–0. 10.1016/j.gpb.2021.09.004 PMC8478109 34560321

[13] Chen Y , Gu S , Chen Y , Lu H , Shi D , Guo J , et al. Six‐month follow‐up of gut microbiota richness in patients with COVID‐19. Gut. 2021;71:222–5. 33833065 10.1136/gutjnl-2021-324090 PMC8666823

[14] Nalbandian A , Sehgal K , Gupta A , Madhavan MV , McGroder C , Stevens JS , et al. Post‐acute COVID‐19 syndrome. Nat Med. 2021;27:601–15. 33753937 10.1038/s41591-021-01283-z PMC8893149

[15] Huang L , Yao Q , Gu X , Wang Q , Ren L , Wang Y , et al. 1‐year outcomes in hospital survivors with COVID‐19: a longitudinal cohort study. Lancet. 2021;398:747–58. 34454673 10.1016/S0140-6736(21)01755-4 PMC8389999

[16] Huang C , Huang L , Wang Y , Li X , Ren L , Gu X , et al. 6‐month consequences of COVID‐19 in patients discharged from hospital: a cohort study. Lancet. 2021;397:220–32. 33428867 10.1016/S0140-6736(20)32656-8 PMC7833295

[17] Liu Q , Mak JWY , Su Q , Yeoh YK , Lui GC , Ng SSS , et al. Gut microbiota dynamics in a prospective cohort of patients with post‐acute COVID‐19 syndrome. Gut. 2022;71:544–52. 35082169 10.1136/gutjnl-2021-325989

[18] Lu J , Peng J , Xiong Q , Liu Z , Lin H , Tan X , et al. Clinical, immunological and virological characterization of COVID‐19 patients that test re‐positive for SARS‐CoV‐2 by RT‐PCR. EBioMedicine. 2020;59:102960. 32853988 10.1016/j.ebiom.2020.102960 PMC7444471

[19] Magne F , Gotteland M , Gauthier L , Zazueta A , Pesoa S , Navarrete P , et al. The Firmicutes/Bacteroidetes ratio: a relevant marker of gut dysbiosis in obese patients? Nutrients. 2020;12:1474. 32438689 10.3390/nu12051474 PMC7285218

[20] Lloyd‐Price J , Mahurkar A , Rahnavard G , Crabtree J , Orvis J , Hall AB , et al. Strains, functions and dynamics in the expanded Human Microbiome Project. Nature. 2017;550:61–6. 28953883 10.1038/nature23889 PMC5831082

[21] Dang AT , Marsland BJ . Microbes, metabolites, and the gut–lung axis. Mucosal Immunol. 2019;12:843–50. 30976087 10.1038/s41385-019-0160-6

[22] Kawabata S , Nagayama R , Hirata M , Shigenaga T , Agarwala KL , Saito T , et al. Tachycitin, a small granular component in horseshoe crab hemocytes, is an antimicrobial protein with chitin‐binding activity. J Biochem. 1996;120:1253–60. 9010778 10.1093/oxfordjournals.jbchem.a021549

[23] Bublitz M , Polle L , Holland C , Heinz DW , Nimtz M , Schubert WD . Structural basis for autoinhibition and activation of Auto, a virulence‐associated peptidoglycan hydrolase of *Listeria monocytogenes* . Mol Microbiol. 2009;71:1509–22. 19210622 10.1111/j.1365-2958.2009.06619.x

[24] Haahtela T , Holgate S , Pawankar R , Akdis CA , Benjaponpitak S , Caraballo L , et al. The biodiversity hypothesis and allergic disease: world allergy organization position statement. World Allergy Organ J. 2013;6:3. 23663440 10.1186/1939-4551-6-3 PMC3646540

[25] Bello MGD , Knight R , Gilbert JA , Blaser MJ . Preserving microbial diversity. Science. 2018;362:33–4. 30287652 10.1126/science.aau8816

[26] Chen Y , Gu S , Chen Y , Lu H , Shi D , Guo J , et al. Six‐month follow‐up of gut microbiota richness in patients with COVID‐19. Gut. 2021;7:222–5. 10.1136/gutjnl-2021-324090 PMC8666823 33833065

[27] Yeoh YK , Zuo T , Lui GC‐Y , Zhang F , Liu Q , Li AY , et al. Gut microbiota composition reflects disease severity and dysfunctional immune responses in patients with COVID‐19. Gut. 2021;70:698–706. 33431578 10.1136/gutjnl-2020-323020 PMC7804842

[28] Gao Z , Xu Y , Sun C , Wang X , Guo Y , Qiu S , et al. A systematic review of asymptomatic infections with COVID‐19. J Microbiol Immunol Infect. 2021;54:12–6. 32425996 10.1016/j.jmii.2020.05.001 PMC7227597

[29] Zou L , Ruan F , Huang M , Liang L , Huang H , Hong Z , et al. SARS‐CoV‐2 viral load in upper respiratory specimens of infected patients. N Engl J Med. 2020;382:1177–9. 32074444 10.1056/NEJMc2001737 PMC7121626

[30] Liu Y , Zhang H , Tang X , Jiang X , Yan X , Liu X , et al. Distinct metagenomic signatures in the SARS‐CoV‐2 infection. Front Cell Infect Microbiol. 2021;11:706970. 34926314 10.3389/fcimb.2021.706970 PMC8674698

[31] Mehrotra DV , Janes HE , Fleming TR , Annunziato PW , Neuzil KM , Carpp LN , et al. Clinical endpoints for evaluating efficacy in COVID‐19 vaccine trials. Ann Intern Med. 2021;174:221–8. 33090877 10.7326/M20-6169 PMC7596738

[32] Vinolo MAR , Rodrigues HG , Nachbar RT , Curi R . Regulation of inflammation by short chain fatty acids. Nutrients. 2011;3:858–76. 22254083 10.3390/nu3100858 PMC3257741

[33] Lumley SF , O'Donnell D , Stoesser NE , Matthews PC , Howarth A , Hatch SB , et al. Antibody status and incidence of SARS‐CoV‐2 infection in health care workers. N Engl J Med. 2021;384:533–40. 33369366 10.1056/NEJMoa2034545 PMC7781098

[34] Prorok‐Hamon M , Friswell MK , Alswied A , Roberts CL , Song F , Flanagan PK , et al. Colonic mucosa‐associated diffusely adherent *afaC+ Escherichia coli* expressing *lpfA* and *pks* are increased in inflammatory bowel disease and colon cancer. Gut. 2014;63:761–70. 23846483 10.1136/gutjnl-2013-304739 PMC3995253

[35] Darfeuille‐Michaud A , Neut C , Barnich N , Lederman E , Di Martino P , Desreumaux P , et al. Presence of adherent *Escherichia coli* strains in ileal mucosa of patients with Crohn's disease. Gastroenterology. 1998;115:1405–13. 9834268 10.1016/s0016-5085(98)70019-8

[36] Yong SJ . Long COVID or post‐COVID‐19 syndrome: putative pathophysiology, risk factors, and treatments. Infect Dis. 2021;53:737–54. 10.1080/23744235.2021.1924397 PMC8146298 34024217

[37] Ihekweazu FD , Engevik MA , Ruan W , Shi Z , Fultz R , Engevik KA , et al. *Bacteroides ovatus* promotes IL‐22 production and reduces trinitrobenzene sulfonic acid‐driven colonic inflammation. Am J Pathol. 2021;191:704–19. 33516788 10.1016/j.ajpath.2021.01.009 PMC8027925

[38] Li S , Wang C , Zhang C , Luo Y , Cheng Q , Yu L , et al. Evaluation of the effects of different *Bacteroides vulgatus* strains against DSS‐induced colitis. J Immunol Res. 2021;2021:9117805. 34195297 10.1155/2021/9117805 PMC8181088

[39] Wang C , Xiao Y , Yu L , Tian F , Zhao J , Zhang H , et al. Protective effects of different *Bacteroides vulgatus* strains against lipopolysaccharide‐induced acute intestinal injury, and their underlying functional genes. J Adv Res. 2021;36:27–37. 35127162 10.1016/j.jare.2021.06.012 PMC8799915

[40] Geva‐Zatorsky N , Sefik E , Kua L , Pasman L , Tan TG , Ortiz‐Lopez A , et al. Mining the human gut microbiota for immunomodulatory organisms. Cell. 2017;168:928–43.e11. 28215708 10.1016/j.cell.2017.01.022 PMC7774263

[41] El Kaoutari A , Armougom F , Gordon JI , Raoult D , Henrissat B . The abundance and variety of carbohydrate‐active enzymes in the human gut microbiota. Nat Rev Microbiol. 2013;11:497–504. 23748339 10.1038/nrmicro3050

[42] Hu X , Zhao Y , Yang Y , Gong W , Sun X , Yang L , et al. *Akkermansia muciniphila* improves host defense against influenza virus infection. Front Microbiol. 2020;11:586476. 33603716 10.3389/fmicb.2020.586476 PMC7884316

[43] Labarta‐Bajo L , Gramalla‐Schmitz A , Gerner RR , Kazane KR , Humphrey G , Schwartz T , et al. CD8 T cells drive anorexia, dysbiosis, and blooms of a commensal with immunosuppressive potential after viral infection. Proc Natl Acad Sci USA. 2020;117:24998–5007. 32958643 10.1073/pnas.2003656117 PMC7547153

[44] Sencio V , Machado MG , Trottein F . The lung–gut axis during viral respiratory infections: the impact of gut dysbiosis on secondary disease outcomes. Mucosal Immunol. 2021;14:296–304. 33500564 10.1038/s41385-020-00361-8 PMC7835650

[45] Sokol H , Contreras V , Maisonnasse P , Desmons A , Delache B . Sencio V*, et al*. SARS‐CoV‐2 infection in nonhuman primates alters the composition and functional activity of the gut microbiota. Gut Microbes. 2021;13:1–19. 10.1080/19490976.2021.1893113 PMC7951961 33685349

[46] Campbell C , McKenney PT , Konstantinovsky D , Isaeva OI , Schizas M , Verter J , et al. Bacterial metabolism of bile acids promotes generation of peripheral regulatory T cells. Nature. 2020;581:475–9. 32461639 10.1038/s41586-020-2193-0 PMC7540721

[47] Winkler ES , Shrihari S , Hykes BL, Jr. , Handley SA , Andhey PS , Huang YS , et al. The intestinal microbiome restricts alphavirus infection and dissemination through a bile acid‐type I IFN signaling axis. Cell. 2020;182:901–18. 32668198 10.1016/j.cell.2020.06.029 PMC7483520

[48] Brodin P . Immune determinants of COVID‐19 disease presentation and severity. Nat Med. 2021;27:28–33. 33442016 10.1038/s41591-020-01202-8

[49] Zhang F , Wan Y , Zuo T , Yeoh YK , Liu Q , Zhang L , et al. Prolonged Impairment of short‐chain fatty acid and l‐isoleucine biosynthesis in gut microbiome in patients with COVID‐19. Gastroenterology. 2022;162:548–61.e4. 34687739 10.1053/j.gastro.2021.10.013 PMC8529231

[50] Zuo T , Liu Q , Zhang F , Lui GC , Tso EY , Yeoh YK , et al. Depicting SARS‐CoV‐2 faecal viral activity in association with gut microbiota composition in patients with COVID‐19. Gut. 2021;70:276–84. 32690600 10.1136/gutjnl-2020-322294 PMC7385744

[51] Zhang Y , Guo R , Kim SH , Shah H , Zhang S , Liang JH , et al. SARS‐CoV‐2 hijacks folate and one‐carbon metabolism for viral replication. Nat Commun. 2021;12:1676. 33723254 10.1038/s41467-021-21903-z PMC7960988

[52] Li Y , Luo ZY , Hu YY , Bi YW , Yang JM , Zou WJ , et al. The gut microbiota regulates autism‐like behavior by mediating vitamin B6 homeostasis in EphB6‐deficient mice. Microbiome. 2020;8:120. 32819434 10.1186/s40168-020-00884-z PMC7441571

[53] Kumrungsee T , Zhang P , Chartkul M , Yanaka N , Kato N . Potential role of vitamin B6 in ameliorating the severity of COVID‐19 and its complications. Front Nutr. 2020;7:562051. 33195363 10.3389/fnut.2020.562051 PMC7658555

[54] Meisel E , Efros O , Bleier J , Beit Halevi T , Segal G , Rahav G , et al. Folate levels in patients hospitalized with coronavirus disease 2019. Nutrients. 2021;13:812. 33801194 10.3390/nu13030812 PMC8001221

[55] Engevik MA , Morra CN , Röth D , Engevik K , Spinler JK , Devaraj S , et al. Microbial metabolic capacity for intestinal folate production and modulation of host folate receptors. Front Microbiol. 2019;10:2305. 31649646 10.3389/fmicb.2019.02305 PMC6795088

[56] Calder PC . Nutrition, immunity and COVID‐19. BMJ Nutr Prev Health. 2020;3:74–92. 10.1136/bmjnph-2020-000085 PMC7295866 33230497

[57] Im JH , Je YS , Baek J , Chung MH , Kwon HY , Lee JS . Nutritional status of patients with COVID‐19. Int J Infect Dis. 2020;100:390–3. 32795605 10.1016/j.ijid.2020.08.018 PMC7418699

[58] Ferreira RLU , Sena‐Evangelista KCM , de Azevedo EP , Pinheiro FI , Cobucci RN , Pedrosa LFC . Selenium in human health and gut microflora: bioavailability of selenocompounds and relationship with diseases. Front Nutr. 2021;8:685317. 34150830 10.3389/fnut.2021.685317 PMC8211732

[59] Kasaikina MV , Kravtsova MA , Lee BC , Seravalli J , Peterson DA , Walter J , et al. Dietary selenium affects host selenoproteome expression by influencing the gut microbiota. FASEB J. 2011;25:2492–9. 21493887 10.1096/fj.11-181990 PMC3114522

[60] Martin M . Cutadapt removes adapter sequences from high‐throughput sequencing reads. EMBnet J. 2011;17:10–2.

[61] Partners NGDCMa . Database resources of the National Genomics Data Center in 2020. Nucleic Acids Res. 2020;48:D24–33. 31702008 10.1093/nar/gkz913 PMC7145560

[62] Wang Y , Song F , Zhu J , Zhang S , Yang Y , Chen T , et al. GSA: Genome Sequence Archive. Genomics Proteomics Bioinformatics. 2017;15:14–8. 28387199 10.1016/j.gpb.2017.01.001 PMC5339404

[63] Li D , Liu CM , Luo R , Sadakane K , Lam TW . MEGAHIT: an ultra‐fast single‐node solution for large and complex metagenomics assembly via succinct de Bruijn graph. Bioinformatics. 2015;31:1674–6. 25609793 10.1093/bioinformatics/btv033

[64] Zhu W , Lomsadze A , Borodovsky M . Ab initio gene identification in metagenomic sequences. Nucleic Acids Res. 2010;38:e132. 20403810 10.1093/nar/gkq275 PMC2896542

[65] Fu L , Niu B , Zhu Z , Wu S , Li W . CD‐HIT: accelerated for clustering the next‐generation sequencing data. Bioinformatics. 2012;28:3150–2. 23060610 10.1093/bioinformatics/bts565 PMC3516142

[66] Luo R , Liu B , Xie Y , Li Z , Huang W , Yuan J , et al. SOAPdenovo2: an empirically improved memory‐efficient short‐read de novo assembler. Gigascience. 2012;1:1–18. 23587118 10.1186/2047-217X-1-18 PMC3626529

[67] Huson DH , Auch AF , Qi J , Schuster SC . MEGAN analysis of metagenomic data. Genome Res. 2007;17:377–86. 17255551 10.1101/gr.5969107 PMC1800929

[68] Buchfink B , Xie C , Huson DH . Fast and sensitive protein alignment using DIAMOND. Nat Methods. 2015;12:59–60. 25402007 10.1038/nmeth.3176

[69] Kanehisa M , Araki M , Goto S , Hattori M , Hirakawa M , Itoh M , et al. KEGG for linking genomes to life and the environment. Nucleic Acids Res. 2008;36:D480–4. 18077471 10.1093/nar/gkm882 PMC2238879

[70] Huerta‐Cepas J , Szklarczyk D , Heller D , Hernández‐Plaza A , Forslund SK , Cook H , et al. eggNOG 5.0: a hierarchical, functionally and phylogenetically annotated orthology resource based on 5090 organisms and 2502 viruses. Nucleic Acids Res. 2019;47:D309–14. 30418610 10.1093/nar/gky1085 PMC6324079

[71] Lombard V , Golaconda Ramulu H , Drula E , Coutinho PM , Henrissat B . The carbohydrate‐active enzymes database (CAZy) in 2013. Nucleic Acids Res. 2014;42:D490–5. 24270786 10.1093/nar/gkt1178 PMC3965031

[72] Alcock BP , Raphenya AR , Lau TTY , Tsang KK , Bouchard M , Edalatmand A , et al. CARD 2020: antibiotic resistome surveillance with the comprehensive antibiotic resistance database. Nucleic Acids Res. 2020;48:D517–25. 31665441 10.1093/nar/gkz935 PMC7145624

[73] Liu B , Zheng D , Jin Q , Chen L , Yang J . VFDB 2019: a comparative pathogenomic platform with an interactive web interface. Nucleic Acids Res. 2019;47:D687–92. 30395255 10.1093/nar/gky1080 PMC6324032

[74] Segata N , Izard J , Waldron L , Gevers D , Miropolsky L , Garrett WS , et al. Metagenomic biomarker discovery and explanation. Genome Biol. 2011;12:R60. 21702898 10.1186/gb-2011-12-6-r60 PMC3218848

[75] Ramette A . Multivariate analyses in microbial ecology. FEMS Microbiol Ecol. 2007;62:142–60. 17892477 10.1111/j.1574-6941.2007.00375.x PMC2121141

[76] Bray JR , Curtis JT . An ordination of upland forest communities of southern Wisconsin. Ecol Monograph. 1957;27:325–49.

[77] Barker M , Rayens W . Partial least squares for discrimination. J Chemom. 2003;17:166–73.

[78] Emiola A , Oh J . High throughput in situ metagenomic measurement of bacterial replication at ultra‐low sequencing coverage. Nat Commun. 2018;9:4956. 30470746 10.1038/s41467-018-07240-8 PMC6251912

[79] Caporaso JG , Kuczynski J , Stombaugh J , Bittinger K , Bushman FD , Costello EK , et al. QIIME allows analysis of high‐throughput community sequencing data. Nat Methods. 2010;7:335–6. 20383131 10.1038/nmeth.f.303 PMC3156573

