# Supplementary material for: Distinct gut microbiota and health outcomes in asymptomatic infection, viral nucleic acid test re‐positive, and convalescent COVID‐19 cases
Source: mLife. 2022 Jun 15;1(2):183–97. doi: 10.1002/mlf2.12022 (PMC9349603; doi:10.1002/mlf2.12022)
Supplement: Supplementary file 1 — Supporting information. [file MLF2-1-183-s001.pdf]

Supplementary figures

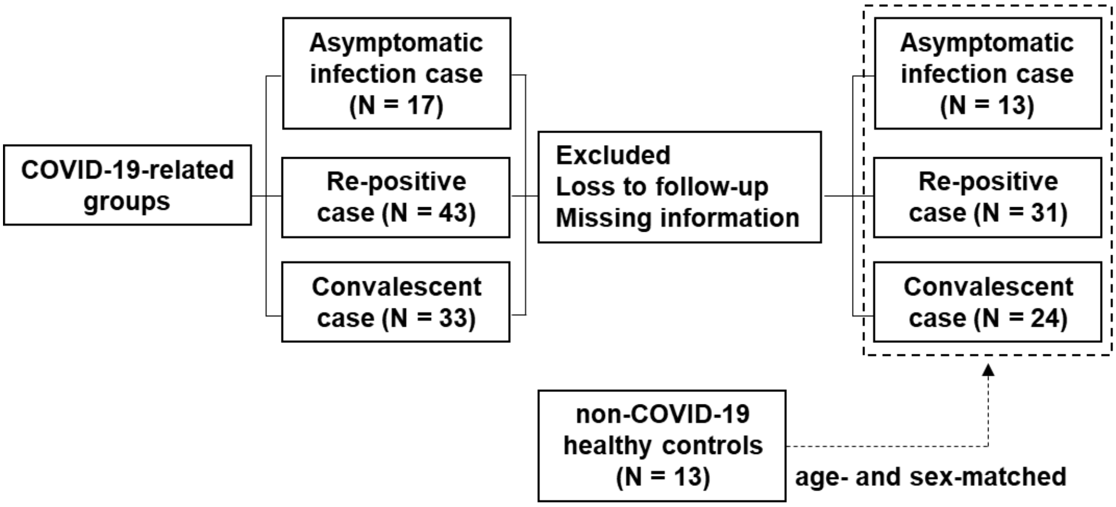

Figure S1 Schematic drawing of the volunteers recruitment

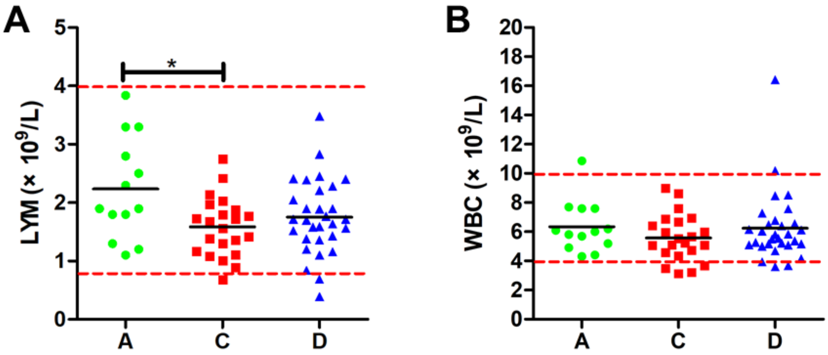

Figure S2 The counts of lymphocyte (LYM) and white blood cell (WBC) in the blood of patients in group A, C and D.

(A) The counts of LYM in the blood of patients in group A (N = 13), C (N = 22) and D (N = 30). The normal reference value is 0.8-4 ($\times 10^9$/L) as the red line shown. There was one case and two cases lower than the normal reference value in group C and D, respectively. (B) The counts of WBC in the blood of patients in group A (N = 13), C (N = 22) and D (N = 30). The normal reference value is 4-10 ($\times 10^9$/L) as the red line shown. One case in group A was higher than the normal reference value, and four cases in group C were lower than the normal reference value. Two cases were higher while three cases were lower than the normal reference value. One-way ANOVA with the least significant difference (LSD) test was used to analyze differences among the groups. Data were shown as mean. *$p < 0.05$.

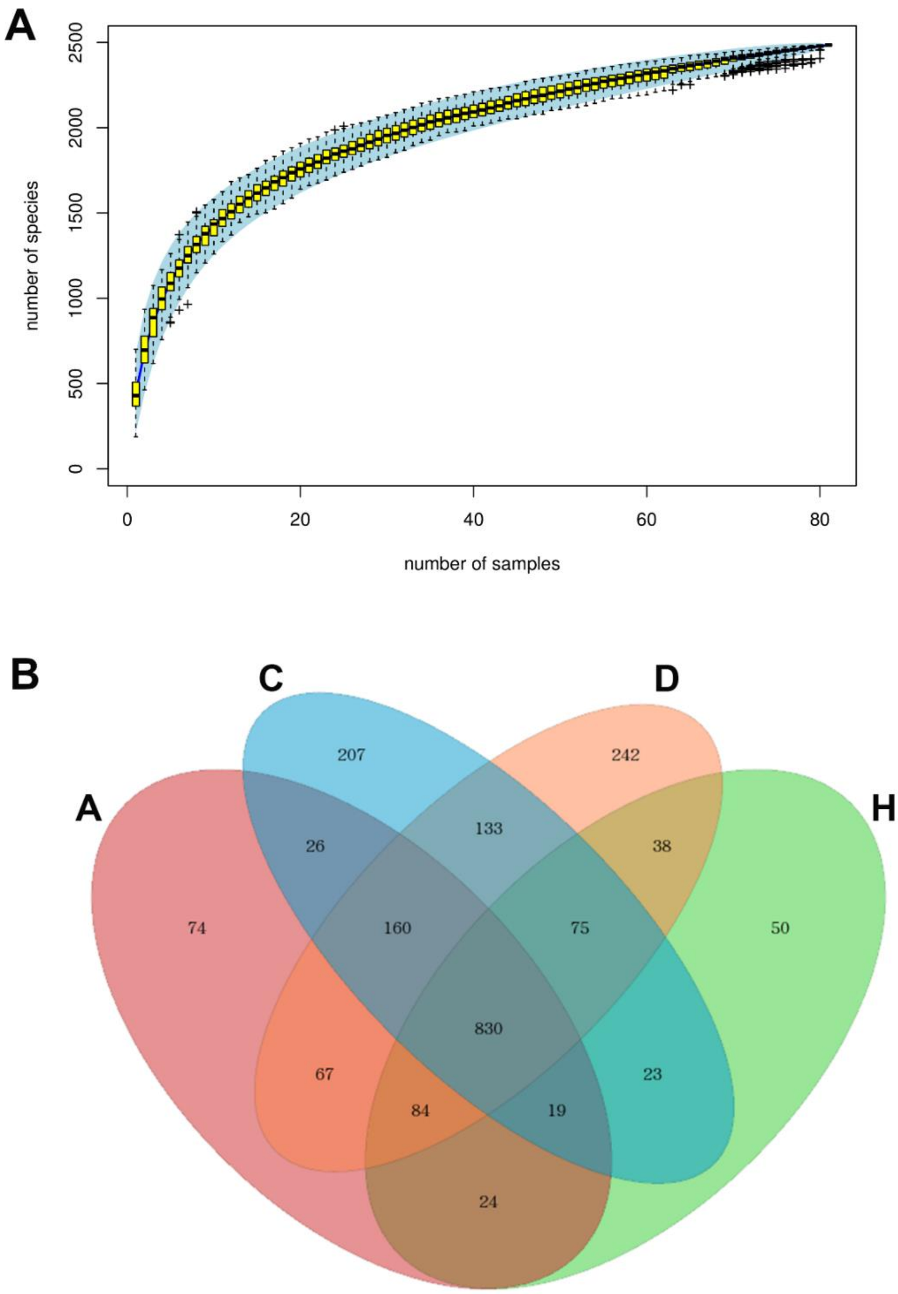

Figure S3 Species accumulation curves and Venn diagram of the number of shared species in groups

(A) Species accumulation curves. The plot showed that adding a new sample increased a few new microbial species to the dataset, indicating the sampling size and sequencing depth were sufficient to represent the overall gut microbial diversity. (B) The venn diagram showed 830 species were shared to all groups, while 74, 207, 242 and 50 species were unique to the group A, C, D and H, respectively.

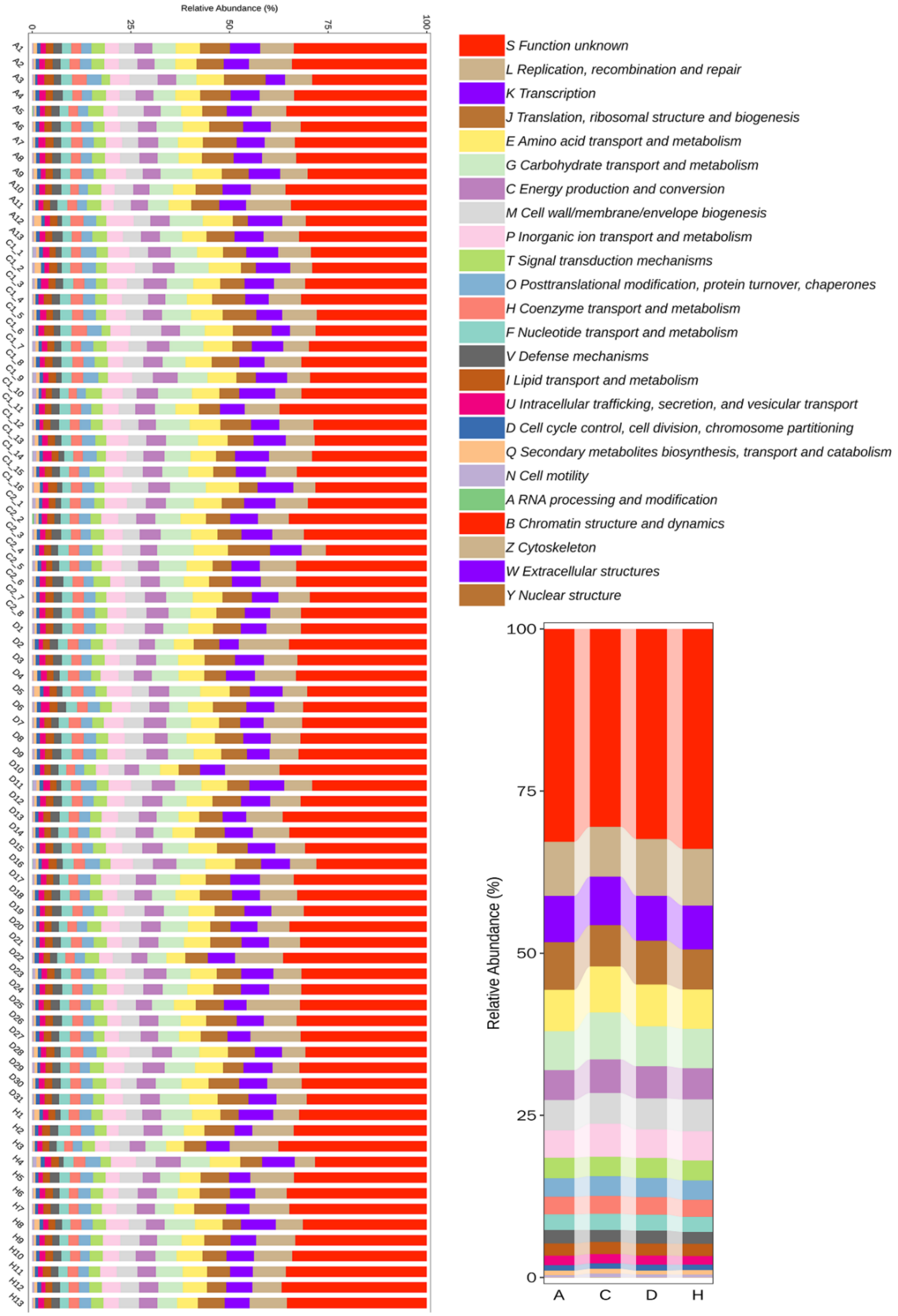

Figure S4 The profile of bacterial orthologous groups based on eggNOG annotations

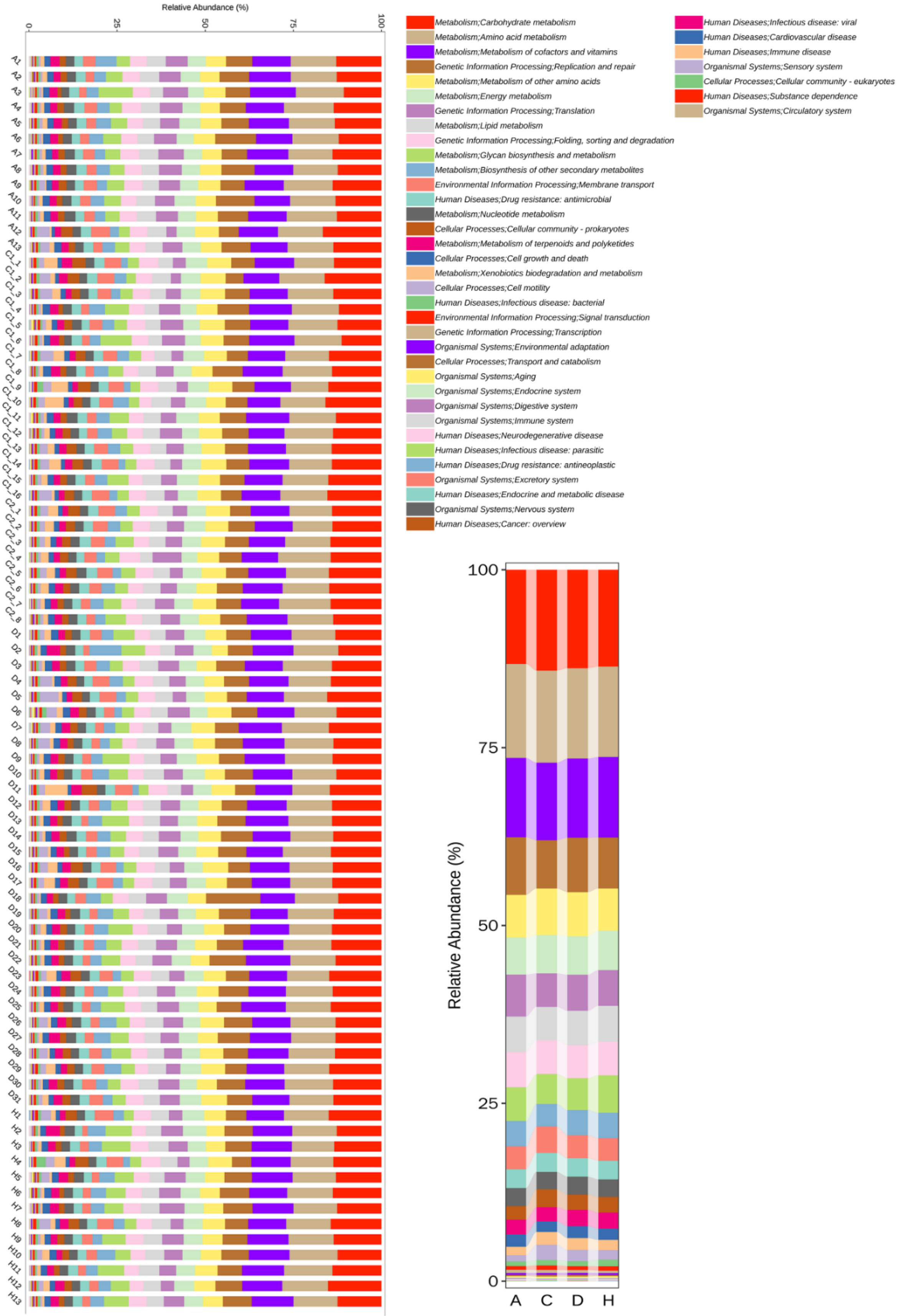

Figure S5 The profile of bacterial metabolic pathway based on KO annotations

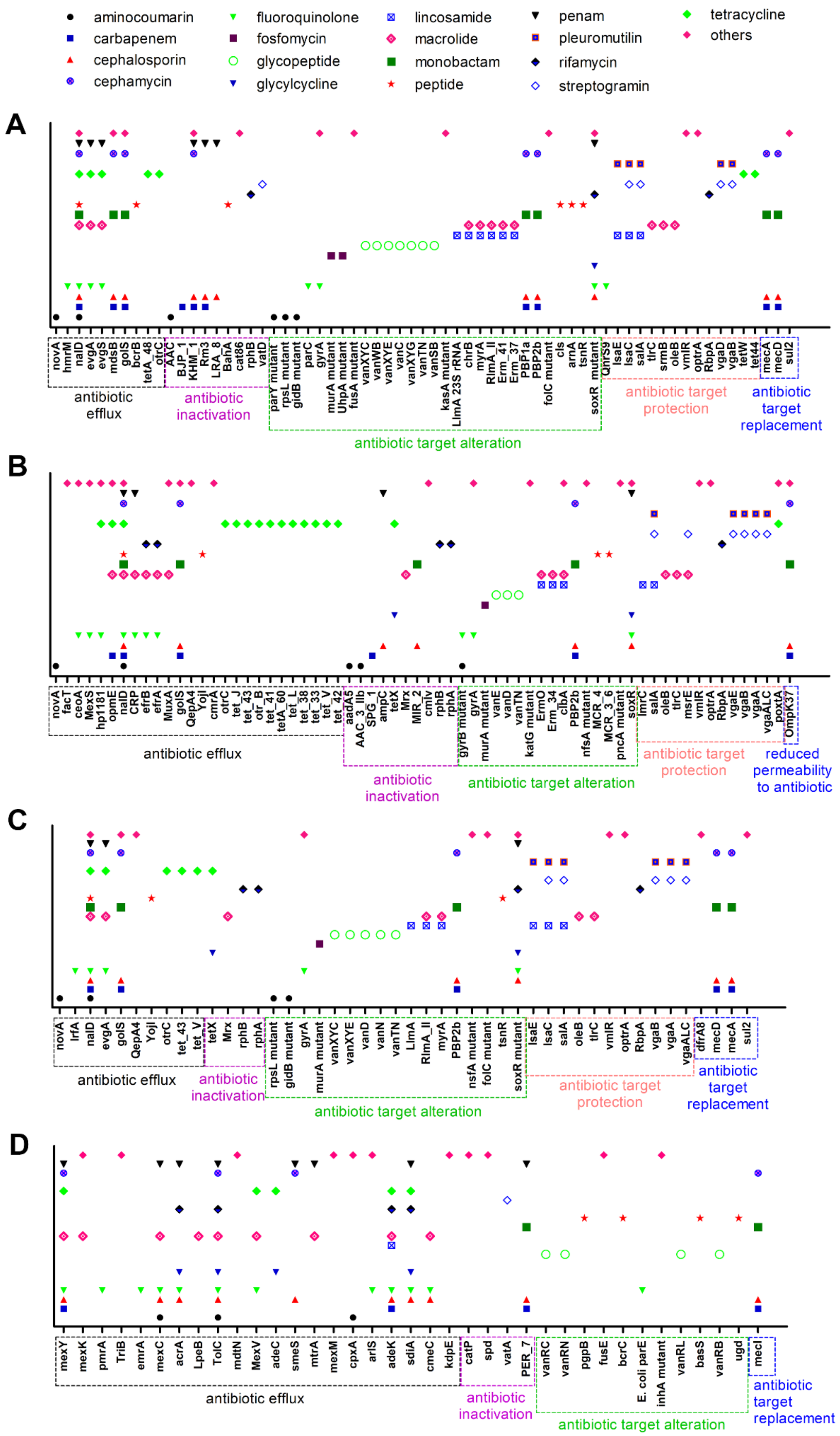

Figure S6 The enriched resistance genes of gut microbiota in COVID-19-related groups

(A) In group A, 67 resistance genes were mainly related to cephalosporin resistance (11), macrolide resistance (11), carbapenem resistance (10), lincosamide resistance (9), fluoroquinolone resistance (8) and cephamycin resistance (8). (B) In group C, 66 antibiotic resistance genes were mainly related to tetracycline resistance (16), macrolide resistance (13), fluoroquinolone resistance (10), cephalosporin resistance (7), carbapenem resistance (6) and streptogramin resistance (6). (C) In group D, 46 antibiotic resistance genes were mainly related to macrolide resistance (7), cephalosporin resistance (6), lincosamide resistance (6), tetracycline resistance (6), pleuromutilin resistance (6). (D) In group H, 37 antibiotic resistance genes were mainly related to fluoroquinolone resistance (12), cephalosporin resistance (10), macrolide resistance (9) and penam resistance (8).

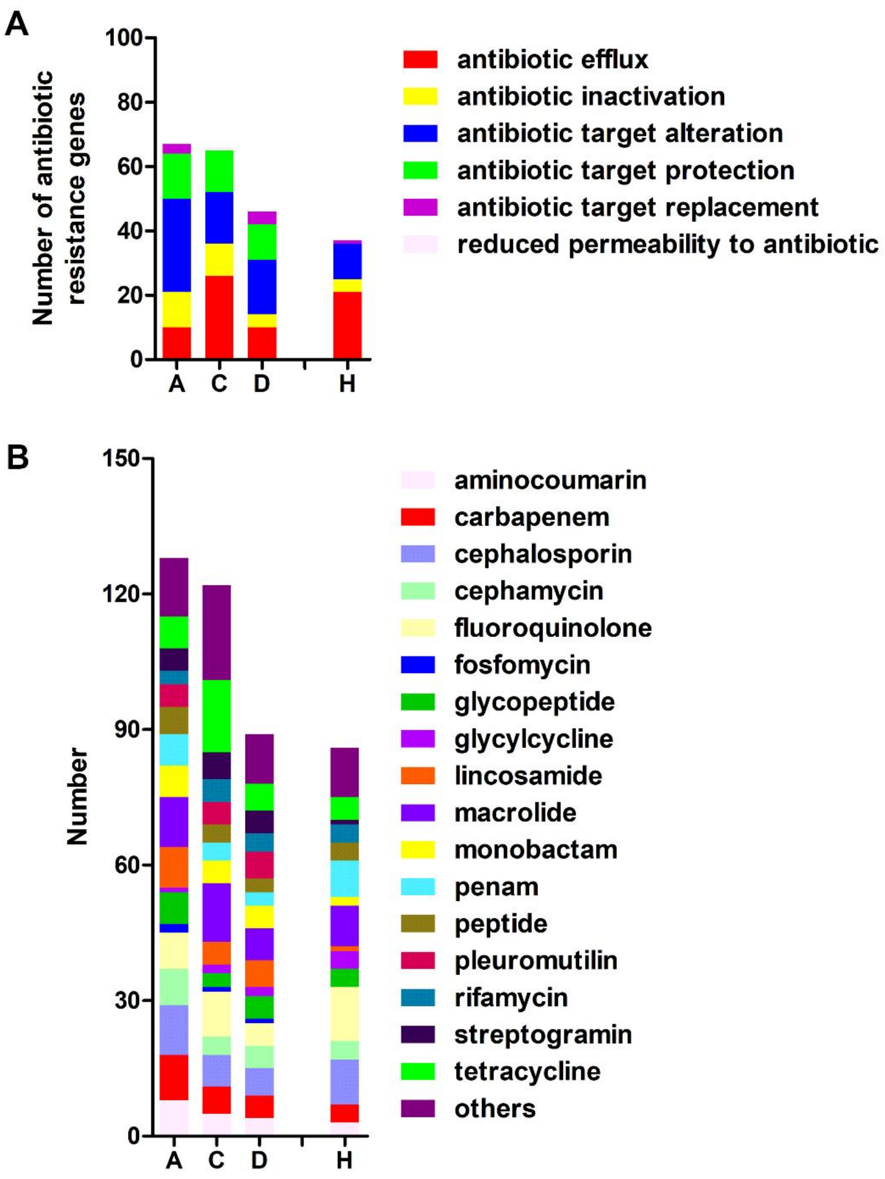

Figure S7 The resistance mechanism and resistant spectrum of resistance genes in COVID-19-related groups

(A) The resistance mechanism of the resistance genes in group A, C, D and H. (B) The resistant spectrum of the resistance genes in group A, C D and H.

Supplementary tables

Table S1 Enriched species in groups using LEfSe analysis

Abundace	Group	LDA_score	P value	Species
4.839998	A	4.443891	0.039843	s__Akkermansia_muciniphila
3.754537	A	3.572035	0.03227	s__Bacteroides_intestinalis
4.429785	A	4.09647	0.002279	s__Bifidobacterium_longum
3.950202	A	3.658459	0.000246	s__Blautia_obeum
2.369887	A	3.1962	0.019203	s__Blautia_sp_AF19_10LB
3.244315	A	3.032211	0.015762	s__Blautia_sp_OM05_6
2.733603	A	3.206317	0.020064	s__Butyricicoccus_sp_OM06_6AC
2.44805	A	3.08765	0.015762	s__Collinsella_sp_AF15_51
3.01922	A	3.116847	0.015432	s__Collinsella_sp_AF37_9
3.083677	A	3.028075	0.043054	s__Collinsella_sp_AM34_10
3.566745	A	3.269161	0.014855	s__Dorea_formicigenerans
3.837781	A	3.426499	0.03332	s__Dorea_longicatena
3.582947	A	3.297046	0.004546	s__Eubacterium_ramulus
2.908623	A	3.015892	0.013272	s__Eubacterium_ventriosum
4.886772	A	4.53395	0.001611	s__Faecalibacterium_prausnitzii
3.791094	A	3.448626	0.013911	s__Faecalibacterium_sp_AF10_46
3.772427	A	3.416373	0.041563	s__Faecalibacterium_sp_AF27_11BH
4.323421	A	3.998531	0.006068	s__Gemmiger_formicilis
2.453205	A	3.102193	0.030426	s__Gordonibacter_pamelaeae
2.499457	A	3.42723	0.011835	s__Gordonibacter_urolithinfaciens
2.233273	A	3.039457	0.001209	s__Intestinibacillus_sp_Marseille_P4005
2.312272	A	3.467289	0.01082	s__Lachnoclostridium_sp_SNUG30386
3.973137	A	3.43352	0.042802	s__Parabacteroides_merdae
2.759748	A	3.478293	0.002818	s__Porphyromonas_asaccharolytica
3.842792	A	3.515797	0.020253	s__Roseburia_hominis
2.50661	A	3.001223	0.003657	s__Roseburia_sp_OM02_15
2.526415	A	3.136858	0.048768	s__Rothia_mucilaginosa
3.139854	A	3.128137	0.017517	s__Ruminococcus_sp_AM28_41
3.668754	A	3.207289	0.005192	s__Ruminococcus_sp_AM42_11
4.142164	A	3.904753	0.023528	s__Ruminococcus_sp_TF11_2AC
2.483736	A	3.360882	0.003976	s__Streptococcus_oralis
2.644429	A	3.2091	0.011835	s__Streptococcus_thermophilus
4.585244	A	4.278783	0.017097	s__Subdoligranulum_sp_APC924_74
3.618943	A	3.261579	0.002655	s__Subdoligranulum_sp_OF01_18
4.720056	C	4.462661	0.004115	s__Akkermansia_muciniphila
4.449554	C	4.128709	0.000622	s__Bifidobacterium_breve
4.608035	C	4.220976	0.000623	s__Bifidobacterium_dentium
2.993554	C	3.022561	0.000483	s__Chitinophaga_sp_K20C18050901
2.10224	C	3.457113	0.034187	s__Clostridium_neonatale
3.056729	C	3.237777	0.003624	s__Collinsella_sp_AM20_15AC
2.699692	C	3.171003	0.026521	s__Coprobacillus_sp_AF09_1A

3.587505	C	3.240345	0.006415	s__Erysipelatoclostridium_ramosum
5.324841	C	4.828871	0.005643	s__Escherichia_coli
2.650063	C	3.093711	0.017694	s__Faecalibacterium_sp_AF10_46
3.120956	C	3.030823	0.011624	s__Faecalibacterium_sp_AM43_5AT
3.343735	C	3.092423	0.008541	s__Faecalibacterium_sp_OF03_6AC
4.49666	C	4.19139	0.028151	s__Hungatella_hathewayi
2.204772	C	3.112719	0.034187	s__Lactobacillus_johnsonii
2.293041	C	3.539852	0.013438	s__Lactobacillus_pantheris
3.31278	C	3.002459	0.014882	s__Megasphaera_micronuciformis
3.297391	C	3.108299	0.004623	s__Merdimonas_faecis
2.97539	C	3.020376	0.015963	s__Pediococcus_acidilactici
4.499115	C	4.132586	0.002642	s__Raoultella_ornithinolytica
3.643284	C	3.400533	0.018162	s__Ruminococcus_sp_AM58_7XD
3.186592	C	3.006158	0.000231	s__Streptococcus_thermophilus
4.377102	D	4.118407	0.018355	s__Akkermansia_muciniphila
3.195676	D	3.467738	0.031326	s__Bacteroides_neonati
2.792276	D	3.010499	0.010517	s__Butyricicoccus_pullicaecorum
3.476324	D	3.412802	0.048136	s__Dysgonomonas_capnocytophagoides
3.449465	D	3.153677	0.023797	s__Eubacterium_ramulus
3.395212	D	3.281265	0.013127	s__Fusobacterium_ulcerans
3.50117	D	3.311529	0.04674	s__Lactobacillus_amylovorus
3.873314	D	3.366469	0.008503	s__Phascolarctobacterium_faecium
3.375136	D	3.12764	0.047075	s__Streptococcus_pasteurianus
3.333216	D	3.133706	9.99E-05	s__Streptococcus_thermophilus
3.710953	D	3.346291	0.009588	s__Subdoligranulum_sp_OF01_18
2.257619	D	3.130501	0.026292	s__Weissella_confusa
3.741143	H	3.40028	0.004526	s__[Eubacterium_]eligens
4.108015	H	3.671282	0.002868	s__Bacteroides_ovatus
4.708685	H	4.337105	0.002099	s__Bacteroides_uniformis
4.983008	H	4.553459	0.012679	s__Bacteroides_vulgatus
3.238758	H	3.034634	0.030237	s__Butyricicoccus_sp_OM04_18BH
3.550357	H	3.431429	0.001051	s__Clostridium_sp_AF27_2AA
2.481864	H	3.099497	0.00311	s__Clostridium_sp_AF34_10BH
2.426802	H	3.160013	0.00241	s__Clostridium_sp_AF36_18BH
2.652995	H	3.390992	0.015004	s__Clostridium_sp_AF50_3
3.728171	H	3.381717	0.000307	s__Clostridium_sp_AM22_11AC
3.958005	H	3.596524	0.003674	s__Clostridium_sp_AM32_2
2.63049	H	3.417187	0.00086	s__Clostridium_sp_OF09_10
3.545307	H	3.192364	0.011674	s__Clostridium_sp_OM04_12AA
3.766387	H	3.415358	0.000742	s__Clostridium_sp_TM06_18
3.279899	H	3.08396	0.000169	s__Desulfotomaculum_sp_OF05_3
3.355616	H	3.161151	0.019377	s__Odoribacter_splanchnicus
4.425793	H	4.082641	0.012107	s__Prevotella_sp_TF12_30
3.379662	H	3.252113	0.021547	s__Sutterella_wadsworthensis

Table S2 Fast-growing species in groups

Group	Species	Average of growth rate	p value
A	s__Bifidobacterium_longum	1.4455	0.000
A	s__Blautia_obeum	1.4450	0.004
A	s__Faecalibacterium_prausnitzii	1.1777	0.018
A	s__Faecalibacterium_sp_AF10_46	1.4485	0.000
A	s__Roseburia_hominis	1.0292	0.041
A	s__Streptococcus_thermophilus	1.2208	0.008
C	s__Akkermansia_muciniphila	1.1580	0.027
C	s__Erysipelatoclostridium_ramosum	1.1333	0.000
C	s__Escherichia_coli	1.4142	0.000
C	s__Faecalibacterium_sp_AF10_46	1.1152	0.010
C	s__Raoultella_ornithinolytica	2.6273	0.000
C	s__Citrobacter_freundii	2.2305	0.001
C	s__Streptococcus_thermophilus	1.2217	0.000
D	s__Akkermansia_muciniphila	1.1350	0.001
D	s__Phascolarctobacterium_faecium	1.4378	0.000
D	s__Streptococcus_thermophilus	1.2131	0.000
H	s__Bacteroides_ovatus	1.1483	0.007
H	s__Bacteroides_vulgatus	1.09	0.005
H	s__Clostridium_sp_AM32_2	1.4862	0.001

Table S3 Enriched bacterial orthologous groups based on eggNOG annotations using LEfSe analysis

Taxa	Abundace	Group	LDA_score	p value
L1__Cellular_Processes_and_Signaling	4.5443	A	3.6384	0.0171
L1__Cellular_Processes_and_Signaling|L2__V_Defense_mechanisms	3.9303	A	3.1593	0.0038
L1__Information_Storage_and_Processing|L2__K_Transcription	3.9899	A	3.1130	0.0096
L1__Cellular_Processes_and_Signaling|L2__U_Intracellular_trafficking_secretion_and_vesicular_transport	3.7290	A	3.0567	0.0257
L1__Information_Storage_and_Processing|L2__L_Replication_recombination_and_repair|L3__ENOG4105C6I	3.5277	A	2.9948	0.0171
L1__Others|L2__S_Function_unknown|L3__ENOG4105WR4	3.2230	A	2.8807	0.0378
L1__Cellular_Processes_and_Signaling|L2__V_Defense_mechanisms|L3__ENOG4105BZ1	3.4147	A	2.8088	0.0009
L1__Others|L2__S_Function_unknown|L3__ENOG4105K5W	3.3484	A	2.7943	0.0293
L1__Information_Storage_and_Processing|L2__K_Transcription|L3__ENOG41067QW	3.3357	A	2.7931	0.0027
L1__Cellular_Processes_and_Signaling|L2__U_Intracellular_trafficking_secretion_and_vesicular_transport|L3__ENOG4105E80	3.2754	A	2.7104	0.0333
L1__Cellular_Processes_and_Signaling|L2__V_Defense_mechanisms|L3__ENOG4105CQU	3.0637	A	2.6750	0.0019
L1__Metabolism|L2__P_Inorganic_ion_transport_and_metabolism|L3__ENOG4108JJB	2.9033	A	2.6735	0.0006
L1__Information_Storage_and_Processing|L2__J_Translation_ribosomal_structure_and_biogenesis|L3__ENOG41082SA	2.9105	A	2.6193	0.0004
L1__Others|L2__S_Function_unknown|L3__ENOG4105VPX	2.9197	A	2.5852	0.0019
L1__Cellular_Processes_and_Signaling|L2__V_Defense_mechanisms|L3__ENOG4105CJ1	2.9627	A	2.5747	0.0016
L1__Information_Storage_and_Processing|L2__L_Replication_recombination_and_repair|L3__ENOG4105F8B	2.9399	A	2.5636	0.0038
L1__Cellular_Processes_and_Signaling|L2__U_Intracellular_trafficking_secretion_and_vesicular_transport|L3__ENOG4105MU5	3.1371	A	2.5488	0.0257
L1__Cellular_Processes_and_Signaling|L2__V_Defense_mechanisms|L3__ENOG4107QJQ	3.1378	A	2.5357	0.0378
L1__Cellular_Processes_and_Signaling|L2__T_Signal_transduction_mechanisms|L3__ENOG4105CK6	3.1609	A	2.5318	0.0011
L1__Information_Storage_and_Processing|L2__J_Translation_ribosomal_structure_and_biogenesis|L3__ENOG4105KAR	2.9765	A	2.5070	0.0196
L1__Information_Storage_and_Processing|L2__J_Translation_ribosomal_structure_and_biogenesis|L3__ENOG4108UNN	2.8183	A	2.4957	0.0032
L1__Metabolism|L2__P_Inorganic_ion_transport_and_metabolism|L3__ENOG4105VRP	2.8903	A	2.4835	0.0096
L1__Cellular_Processes_and_Signaling|L2__U_Intracellular_trafficking_secretion_and_vesicular_transport|L3__ENOG4105DTV	2.9685	A	2.4691	0.0225
L1__Metabolism|L2__C_Energy_production_and_conversion|L3__ENOG4105KNM	2.8514	A	2.4658	0.0149
L1__Metabolism|L2__P_Inorganic_ion_transport_and_metabolism|L3__ENOG4105C59	3.0385	A	2.4636	0.0019
L1__Information_Storage_and_Processing|L2__K_Transcription|L3__ENOG4108ZF1	2.8914	A	2.4601	0.0002
L1__Information_Storage_and_Processing|L2__L_Replication_recombination_and_repair|L3__ENOG4108RZS	2.9493	A	2.4585	0.0171
L1__Cellular_Processes_and_Signaling|L2__T_Signal_transduction_mechanisms|L3__ENOG4108YZM	2.9220	A	2.4569	0.0378
L1__Others|L2__S_Function_unknown|L3__ENOG4105ZN2	2.8389	A	2.4522	0.0378

L1__Information_Storage_and_Processing|L2__J_Translation_ribosomal_structure_and_biogenesis|L3__ENOG4105BZT	2.8228	A	2.4508	0.0014
L1__Others|L2__S_Function_unknown|L3__ENOG4105KGF	2.8492	A	2.4478	0.0083
L1__Information_Storage_and_Processing|L2__K_Transcription|L3__ENOG4105KST	2.8825	A	2.4475	0.0061
L1__Information_Storage_and_Processing|L2__L_Replication_recombination_and_repair|L3__ENOG4105C77	2.9904	A	2.4411	0.0225
L1__Others|L2__S_Function_unknown|L3__ENOG4105VB6	2.8572	A	2.4309	0.0225
L1__Others|L2__S_Function_unknown|L3__ENOG4105KRY	2.8639	A	2.4238	0.0171
L1__Metabolism|L2__P_Inorganic_ion_transport_and_metabolism|L3__ENOG4105CE1	2.8077	A	2.4180	0.0378
L1__Information_Storage_and_Processing|L2__K_Transcription|L3__ENOG4108VZQ	2.9496	A	2.4173	0.0483
L1__Cellular_Processes_and_Signaling|L2__M_Cell_wall_membrane_envelope_biogenesis|L3__ENOG4105K64	2.9338	A	2.4170	0.0083
L1__Cellular_Processes_and_Signaling|L2__O_Posttranslational_modification_protein_turnover_chaperones|L3__ENOG4105C2Z	2.8774	A	2.4144	0.0171
L1__Information_Storage_and_Processing|L2__K_Transcription|L3__ENOG4105VZN	2.8325	A	2.4072	0.0333
L1__Others|L2__S_Function_unknown|L3__ENOG4105WSQ	2.8340	A	2.4068	0.0293
L1__Others|L2__S_Function_unknown|L3__ENOG4108Z5G	2.8909	A	2.4000	0.0149
L1__Others|L2__S_Function_unknown|L3__ENOG4105CYH	2.9299	A	2.3961	0.0378
L1__Information_Storage_and_Processing|L2__J_Translation_ribosomal_structure_and_biogenesis|L3__ENOG4105K5M	3.0452	A	2.3904	0.0196
L1__Information_Storage_and_Processing|L2__J_Translation_ribosomal_structure_and_biogenesis|L3__ENOG4108UJY	2.7974	A	2.3793	0.0378
L1__Information_Storage_and_Processing|L2__J_Translation_ribosomal_structure_and_biogenesis|L3__ENOG4108YZX	2.8612	A	2.3590	0.0428
L1__Cellular_Processes_and_Signaling|L2__V_Defense_mechanisms|L3__ENOG4105C3S	3.0677	A	2.3554	0.0428
L1__Information_Storage_and_Processing|L2__J_Translation_ribosomal_structure_and_biogenesis|L3__ENOG4108Z04	2.8914	A	2.3432	0.0483
L1__Information_Storage_and_Processing|L2__J_Translation_ribosomal_structure_and_biogenesis|L3__ENOG4105VCC	2.8062	A	2.3377	0.0378
L1__Cellular_Processes_and_Signaling|L2__M_Cell_wall_membrane_envelope_biogenesis|L3__ENOG4105DZ1	2.8270	A	2.3173	0.0096
L1__Information_Storage_and_Processing|L2__J_Translation_ribosomal_structure_and_biogenesis|L3__ENOG4108ZDX	2.8174	A	2.2899	0.0483
L1__Cellular_Processes_and_Signaling|L2__O_Posttranslational_modification_protein_turnover_chaperones|L3__ENOG4105C3M	2.7720	A	2.2794	0.0023
L1__Metabolism|L2__E_Amino_acid_transport_and_metabolism|L3__ENOG4105CDA	2.9783	A	2.2067	0.0333
L1__Metabolism	4.5918	C	3.1974	0.0131
L1__Metabolism|L2__E_Amino_acid_transport_and_metabolism	4.0527	C	3.0209	0.0012
L1__Others|L2__S_Function_unknown|L3__ENOG410619I	3.6718	C	2.8935	0.0259
L1__Others|L2__S_Function_unknown|L3__ENOG4106D3Q	3.0080	C	2.6593	0.0083
L1__Information_Storage_and_Processing|L2__L_Replication_recombination_and_repair|L3__ENOG4108BUD	2.9416	C	2.5604	0.0131
L1__Cellular_Processes_and_Signaling|L2__V_Defense_mechanisms|L3__ENOG4105BZ1	3.3084	C	2.5265	0.0282
L1__Cellular_Processes_and_Signaling|L2__M_Cell_wall_membrane_envelope_biogenesis|L3__ENOG4107Y68	2.9668	C	2.5165	0.0038

L1__Information_Storage_and_Processing|L2__L_Replication_recombination_and_repair|L3__ENOG4105F2I	2.9477	C	2.5063	0.0386
L1__Information_Storage_and_Processing|L2__K_Transcription|L3__ENOG4105ETE	3.1323	C	2.4361	0.0131
L1__Metabolism|L2__E_Amino_acid_transport_and_metabolism|L3__ENOG4105CDA	3.0644	C	2.4223	0.0025
L1__Cellular_Processes_and_Signaling|L2__M_Cell_wall_membrane_envelope_biogenesis|L3__ENOG4105CNA	2.8544	C	2.4129	0.0239
L1__Metabolism|L2__G_Carbohydrate_transport_and_metabolism|L3__ENOG4105C5Y	2.9829	C	2.4059	0.0046
L1__Cellular_Processes_and_Signaling|L2__V_Defense_mechanisms|L3__ENOG4105CJ1	2.9573	C	2.3946	0.0025
L1__Metabolism|L2__E_Amino_acid_transport_and_metabolism|L3__ENOG4105KNY	2.8818	C	2.3907	0.0025
L1__Metabolism|L2__P_Inorganic_ion_transport_and_metabolism|L3__ENOG4105C2T	3.0558	C	2.3892	0.0109
L1__Metabolism|L2__P_Inorganic_ion_transport_and_metabolism|L3__ENOG4105CJM	3.0150	C	2.3750	0.0031
L1__Information_Storage_and_Processing|L2__L_Replication_recombination_and_repair|L3__ENOG4108UUM	3.0090	C	2.3597	0.0109
L1__Cellular_Processes_and_Signaling|L2__O_Posttranslational_modification_protein_turnover_chaperones|L3__ENOG4105WAZ	2.7977	C	2.3511	0.0156
L1__Metabolism|L2__P_Inorganic_ion_transport_and_metabolism|L3__ENOG4105C2J	3.0208	C	2.3450	0.0386
L1__Metabolism|L2__G_Carbohydrate_transport_and_metabolism|L3__ENOG4105CNN	2.8888	C	2.3087	0.0091
L1__Metabolism|L2__E_Amino_acid_transport_and_metabolism|L3__ENOG4105E5Q	2.7384	C	2.2849	0.0100
L1__Metabolism|L2__E_Amino_acid_transport_and_metabolism|L3__ENOG4105C3U	2.9204	C	2.2768	0.0046
L1__Metabolism|L2__P_Inorganic_ion_transport_and_metabolism|L3__ENOG4108JJB	2.8332	C	2.2748	0.0091
L1__Information_Storage_and_Processing|L2__K_Transcription|L3__ENOG4105E98	2.7893	C	2.2746	0.0005
L1__Metabolism|L2__C_Energy_production_and_conversion|L3__ENOG4107QIW	2.8201	C	2.2712	0.0131
L1__Metabolism|L2__F_Nucleotide_transport_and_metabolism|L3__ENOG4105C2W	2.9068	C	2.2685	0.0042
L1__Metabolism|L2__C_Energy_production_and_conversion|L3__ENOG4105C6N	2.7879	C	2.2591	0.0010
L1__Metabolism|L2__G_Carbohydrate_transport_and_metabolism|L3__ENOG4105CS2	2.7552	C	2.2517	0.0028
L1__Metabolism|L2__E_Amino_acid_transport_and_metabolism|L3__ENOG4105C53	2.8680	C	2.2436	0.0202
L1__Metabolism|L2__G_Carbohydrate_transport_and_metabolism|L3__ENOG4108IJ9	2.7987	C	2.2420	0.0034
L1__Information_Storage_and_Processing|L2__L_Replication_recombination_and_repair|L3__ENOG4105C24	2.7145	C	2.2087	0.0000
L1__Information_Storage_and_Processing|L2__L_Replication_recombination_and_repair|L3__ENOG41067QG	2.8165	C	2.2031	0.0305
L1__Metabolism|L2__G_Carbohydrate_transport_and_metabolism|L3__ENOG4105CMG	2.7243	C	2.2030	0.0259
L1__Metabolism|L2__G_Carbohydrate_transport_and_metabolism|L3__ENOG4105CSH	2.7595	C	2.2023	0.0220
L1__Metabolism|L2__E_Amino_acid_transport_and_metabolism|L3__ENOG4108JQ7	2.7852	C	2.1970	0.0013
L1__Information_Storage_and_Processing|L2__K_Transcription|L3__ENOG4108ZF1	2.7292	C	2.1960	0.0259
L1__Others|L2__S_Function_unknown|L3__ENOG4105KEM	2.7645	C	2.1946	0.0046
L1__Metabolism|L2__C_Energy_production_and_conversion|L3__ENOG4105CCY	2.7271	C	2.1932	0.0038

L1__Cellular_Processes_and_Signaling|L2__O_Posttranslational_modification_protein_turnover_chaperones|L3__ENOG4105C3H	2.7005	C	2.1897	0.0131
L1__Metabolism|L2__P_Inorganic_ion_transport_and_metabolism|L3__ENOG4105C52	2.7951	C	2.1656	0.0100
L1__Metabolism|L2__G_Carbohydrate_transport_and_metabolism|L3__ENOG4105CJQ	2.6905	C	2.1651	0.0486
L1__Information_Storage_and_Processing|L2__J_Translation_ribosomal_structure_and_biogenesis|L3__ENOG4105VCC	2.7428	C	2.1467	0.0062
L1__Cellular_Processes_and_Signaling|L2__M_Cell_wall_membrane_envelope_biogenesis|L3__ENOG4105CJN	2.7084	C	2.1466	0.0016
L1__Cellular_Processes_and_Signaling|L2__O_Posttranslational_modification_protein_turnover_chaperones|L3__ENOG4105C2Z	2.8364	C	2.1453	0.0450
L1__Cellular_Processes_and_Signaling|L2__O_Posttranslational_modification_protein_turnover_chaperones|L3__ENOG4105C0H	2.7090	C	2.1352	0.0386
L1__Metabolism|L2__E_Amino_acid_transport_and_metabolism|L3__ENOG4105C7K	2.7116	C	2.1165	0.0062
L1__Metabolism|L2__E_Amino_acid_transport_and_metabolism|L3__ENOG4105CPQ	2.7642	C	2.1033	0.0051
L1__Cellular_Processes_and_Signaling|L2__M_Cell_wall_membrane_envelope_biogenesis|L3__ENOG4105DZ1	2.7871	C	2.0859	0.0259
L1__Information_Storage_and_Processing|L2__L_Replication_recombination_and_repair|L3__ENOG4105F3K	2.6997	C	2.0321	0.0386
L1__Others|L2__S_Function_unknown|L3__ENOG4105YNI	3.8235	D	3.1945	0.0097
L1__Cellular_Processes_and_Signaling|L2__V_Defense_mechanisms	3.8694	D	2.9109	0.0261
L1__Information_Storage_and_Processing|L2__K_Transcription|L3__ENOG41067QW	3.2438	D	2.6220	0.0018
L1__Others|L2__S_Function_unknown|L3__ENOG410766Y	3.1225	D	2.5779	0.0173
L1__Cellular_Processes_and_Signaling|L2__V_Defense_mechanisms|L3__ENOG4105BZ1	3.3204	D	2.5773	0.0199
L1__Metabolism|L2__P_Inorganic_ion_transport_and_metabolism|L3__ENOG4108JJB	2.8667	D	2.3924	0.0008
L1__Cellular_Processes_and_Signaling|L2__T_Signal_transduction_mechanisms|L3__ENOG4105CK6	3.1338	D	2.3923	0.0015
L1__Information_Storage_and_Processing|L2__K_Transcription|L3__ENOG4108ZF1	2.7949	D	2.3462	0.0030
L1__Cellular_Processes_and_Signaling|L2__V_Defense_mechanisms|L3__ENOG4105CQU	2.9664	D	2.3359	0.0057
L1__Cellular_Processes_and_Signaling|L2__M_Cell_wall_membrane_envelope_biogenesis|L3__ENOG4105DZ1	2.7956	D	2.3181	0.0162
L1__Information_Storage_and_Processing|L2__K_Transcription|L3__ENOG4105KST	2.8113	D	2.3065	0.0078
L1__Metabolism|L2__C_Energy_production_and_conversion|L3__ENOG4105KNM	2.7412	D	2.3010	0.0131
L1__Others|L2__S_Function_unknown|L3__ENOG4108Z5G	2.8252	D	2.2859	0.0384
L1__Others|L2__S_Function_unknown|L3__ENOG4105VPX	2.7361	D	2.2836	0.0186
L1__Information_Storage_and_Processing|L2__J_Translation_ribosomal_structure_and_biogenesis|L3__ENOG4108ZDX	2.7741	D	2.2670	0.0261
L1__Cellular_Processes_and_Signaling|L2__O_Posttranslational_modification_protein_turnover_chaperones|L3__ENOG4105C3M	2.7693	D	2.2669	0.0151
L1__Information_Storage_and_Processing|L2__K_Transcription|L3__ENOG4105VZN	2.7499	D	2.2620	0.0360
L1__Cellular_Processes_and_Signaling|L2__V_Defense_mechanisms|L3__ENOG4105CJ1	2.8795	D	2.2609	0.0084
L1__Others|L2__S_Function_unknown|L3__ENOG4105VDK	2.8001	D	2.2523	0.0131
L1__Others|L2__S_Function_unknown|L3__ENOG4105VB6	2.7510	D	2.2513	0.0162

L1__Information_Storage_and_Processing|L2__J_Translation_ribosomal_structure_and_biogenesis|L3__ENOG4108UIK	2.7156	D	2.2493	0.0162
L1__Metabolism|L2__E_Amino_acid_transport_and_metabolism|L3__ENOG4108JQ7	2.7251	D	2.2435	0.0105
L1__Information_Storage_and_Processing|L2__L_Replication_recombination_and_repair|L3__ENOG4108UUM	2.9688	D	2.2329	0.0279
L1__Metabolism|L2__G_Carbohydrate_transport_and_metabolism|L3__ENOG4105C5Y	2.8845	D	2.2326	0.0261
L1__Metabolism|L2__P_Inorganic_ion_transport_and_metabolism|L3__ENOG4105C59	2.9879	D	2.2309	0.0121
L1__Metabolism|L2__F_Nucleotide_transport_and_metabolism|L3__ENOG4108V82	2.6959	D	2.1856	0.0261
L1__Cellular_Processes_and_Signaling|L2__O_Posttranslational_modification_protein_turnover_chaperones|L3__ENOG4105C2Z	2.8549	D	2.1794	0.0491
L1__Metabolism|L2__E_Amino_acid_transport_and_metabolism|L3__ENOG4105BZG	2.8357	D	2.1728	0.0279
L1__Others|L2__S_Function_unknown|L3__ENOG4108Z66	2.7289	D	2.0109	0.0338

Table S4 Enriched bacterial metabolic pathway based on KO annotations using LEfSe analysis

Taxa	Abundace	Group	LDA_score	Pvalue
L1__Genetic_Information_Processing	5.2904	A	3.9794	0.0129
L1__Genetic_Information_Processing|L2__Translation	4.7750	A	3.6378	0.0378
L1__Metabolism|L2__Amino_acid_metabolism	5.1207	A	3.4410	0.0149
L1__Metabolism|L2__Metabolism_of_other_amino_acids|L3__D_Alanine_metabolism	4.2979	A	3.2479	0.0005
L1__Genetic_Information_Processing|L2__Translation|L3__Aminoacyl_tRNA_biosynthesis	4.2763	A	3.2407	0.0044
L1__Human_Diseases|L2__Drug_resistance_antimicrobial|L3__Vancomycin_resistance	4.1894	A	3.2356	0.0005
L1__Metabolism|L2__Glycan_biosynthesis_and_metabolism|L3__Peptidoglycan_biosynthesis	4.2572	A	3.1526	0.0129
L1__Genetic_Information_Processing|L2__Replication_and_repair|L3__Nucleotide_excision_repair	4.0070	A	3.1250	0.0027
L1__Genetic_Information_Processing|L2__Replication_and_repair|L3__Base_excision_repair	4.1019	A	3.1128	0.0027
L1__Metabolism|L2__Carbohydrate_metabolism|L3__Starch_and_sucrose_metabolism	4.1772	A	3.0800	0.0171
L1__Metabolism|L2__Metabolism_of_cofactors_and_vitamins|L3__Porphyrin_and_chlorophyll_metabolism	3.8740	A	2.9683	0.0257
L1__Cellular_Processes|L2__Cellular_community_prokaryotes|L3__Quorum_sensing	3.8624	A	2.8690	0.0052
L1__Metabolism|L2__Amino_acid_metabolism|L3__Lysine_biosynthesis	4.1361	A	2.8392	0.0378
L1__Metabolism|L2__Amino_acid_metabolism|L3__Cysteine_and_methionine_metabolism	4.0656	A	2.8008	0.0149
L1__Metabolism|L2__Lipid_metabolism|L3__Secondary_bile_acid_biosynthesis	3.7478	A	2.7974	0.0257
L1__Metabolism|L2__Metabolism_of_other_amino_acids|L3__Selenocompound_metabolism	4.0621	A	2.7618	0.0483
L1__Genetic_Information_Processing|L2__Transcription|L3__RNA_polymerase	3.6669	A	2.7513	0.0023
L1__Genetic_Information_Processing|L2__Transcription	3.6673	A	2.7509	0.0023
L1__Metabolism|L2__Amino_acid_metabolism|L3__Arginine_biosynthesis	4.0289	A	2.7069	0.0257
L1__Organismal_Systems|L2__Sensory_system|L3__Olfactory_transduction	0.4017	A	2.6743	0.0338
L1__Organismal_Systems|L2__Sensory_system	0.4017	A	2.6591	0.0338
L1__Organismal_Systems|L2__Immune_system|L3__Complement_and_coagulation_cascades	0.4927	A	2.3543	0.0036
L1__Human_Diseases|L2__Infectious_disease_bacterial|L3__Tuberculosis	3.0706	A	2.3462	0.0181
L1__Cellular_Processes|L2__Transport_and_catabolism|L3__Endocytosis	0.6719	A	2.1896	0.0196
L1__Cellular_Processes|L2__Transport_and_catabolism|L3__Autophagy_yeast	2.3875	A	2.0514	0.0014
L1__Cellular_Processes|L2__Cell_motility	4.3308	C	3.6952	0.0170
L1__Environmental_Information_Processing|L2__Membrane_transport	4.5607	C	3.6054	0.0450
L1__Metabolism|L2__Metabolism_of_other_amino_acids	4.8139	C	3.5455	0.0007
L1__Cellular_Processes|L2__Cell_motility|L3__Flagellar_assembly	4.0076	C	3.4775	0.0143

L1__Cellular_Processes|L2__Cell_motility|L3__Bacterial_chemotaxis	4.0508	C	3.2915	0.0486
L1__Metabolism|L2__Metabolism_of_other_amino_acids|L3__D_Alanine_metabolism	4.2902	C	3.2122	0.0000
L1__Metabolism|L2__Amino_acid_metabolism|L3__Valine_leucine_and_isoleucine_biosynthesis	4.4607	C	3.2043	0.0083
L1__Environmental_Information_Processing|L2__Membrane_transport|L3__ABC_transporters	3.9025	C	3.1180	0.0062
L1__Metabolism|L2__Xenobiotics_biodegradation_and_metabolism|L3__Nitrotoluene_degradation	3.6368	C	3.0556	0.0012
L1__Metabolism|L2__Metabolism_of_other_amino_acids|L3__Selenocompound_metabolism	4.0841	C	2.9021	0.0015
L1__Cellular_Processes|L2__Transport_and_catabolism|L3__Endocytosis	0.5890	C	2.8704	0.0091
L1__Cellular_Processes|L2__Cellular_community_prokaryotes|L3__Quorum_sensing	3.8632	C	2.8575	0.0016
L1__Cellular_Processes|L2__Transport_and_catabolism|L3__Peroxisome	3.2556	C	2.8230	0.0031
L1__Metabolism|L2__Lipid_metabolism|L3__Fatty_acid_degradation	3.6265	C	2.7651	0.0220
L1__Metabolism|L2__Glycan_biosynthesis_and_metabolism|L3__Arabinogalactan_biosynthesis_Mycobacterium	3.2598	C	2.7349	0.0008
L1__Genetic_Information_Processing|L2__Transcription|L3__RNA_polymerase	3.6397	C	2.6337	0.0120
L1__Genetic_Information_Processing|L2__Transcription	3.6399	C	2.6327	0.0120
L1__Metabolism|L2__Lipid_metabolism|L3__Biosynthesis_of_unsaturated_fatty_acids	3.1251	C	2.5873	0.0202
L1__Metabolism|L2__Lipid_metabolism|L3__Glycerolipid_metabolism	3.6992	C	2.5771	0.0450
L1__Metabolism|L2__Xenobiotics_biodegradation_and_metabolism|L3__Aminobenzoate_degradation	3.2232	C	2.5745	0.0170
L1__Metabolism|L2__Amino_acid_metabolism|L3__Tryptophan_metabolism	3.3618	C	2.5334	0.0259
L1__Metabolism|L2__Xenobiotics_biodegradation_and_metabolism|L3__Caprolactam_degradation	3.1353	C	2.4941	0.0417
L1__Human_Diseases|L2__Immune_disease	0.2350	C	2.4847	0.0412
L1__Metabolism|L2__Lipid_metabolism|L3__Glycerophospholipid_metabolism	3.7515	C	2.4617	0.0386
L1__Metabolism|L2__Metabolism_of_terpenoids_and_polyketides|L3__Geraniol_degradation	2.6061	C	2.3430	0.0265
L1__Metabolism|L2__Xenobiotics_biodegradation_and_metabolism|L3__Chlorocyclohexane_and_chlorobenzene_degradation	2.9583	C	2.3376	0.0220
L1__Human_Diseases|L2__Infectious_disease_parasitic	2.8302	C	2.2683	0.0016
L1__Metabolism|L2__Energy_metabolism|L3__Methane_metabolism	3.7063	C	2.1651	0.0100
L1__Human_Diseases|L2__Infectious_disease_parasitic|L3__African_trypanosomiasis	2.4874	C	2.1532	0.0270
L1__Metabolism|L2__Metabolism_of_cofactors_and_vitamins|L3__Retinol_metabolism	2.4042	C	2.1086	0.0318
L1__Genetic_Information_Processing|L2__Translation|L3__Ribosome_biogenesis_in_eukaryotes	2.9153	C	2.0690	0.0075
L1__Human_Diseases|L2__Infectious_disease_bacterial|L3__Staphylococcus_aureus_infection	2.3700	C	2.0473	0.0016
L1__Metabolism|L2__Carbohydrate_metabolism|L3__C5_Branched_dibasic_acid_metabolism	3.7792	D	3.4246	0.0094
L1__Metabolism|L2__Metabolism_of_other_amino_acids|L3__D_Alanine_metabolism	4.2758	D	3.1424	0.0009

L1__Human_Diseases|L2__Drug_resistance_antimicrobial|L3__Vancomycin_resistance	4.1358	D	2.9003	0.0297
L1__Metabolism|L2__Metabolism_of_cofactors_and_vitamins|L3__Porphyrin_and_chlorophyll_metabolism	3.8549	D	2.8576	0.0244
L1__Cellular_Processes|L2__Transport_and_catabolism|L3__Peroxisome	3.2032	D	2.7848	0.0073
L1__Metabolism|L2__Metabolism_of_other_amino_acids|L3__Selenocompound_metabolism	4.0598	D	2.7056	0.0173
L1__Cellular_Processes|L2__Cellular_community_prokaryotes|L3__Quorum_sensing	3.8277	D	2.6062	0.0435
L1__Metabolism|L2__Glycan_biosynthesis_and_metabolism|L3__Arabinogalactan_biosynthesis_Mycobacterium	3.1199	D	2.4005	0.0261
L1__Genetic_Information_Processing|L2__Transcription	3.6192	D	2.3941	0.0228
L1__Genetic_Information_Processing|L2__Transcription|L3__RNA_polymerase	3.6181	D	2.3887	0.0228
L1__Metabolism|L2__Metabolism_of_other_amino_acids|L3__D_Arginine_and_D_ornithine_metabolism	2.9080	D	2.3321	0.0090
L1__Metabolism|L2__Xenobiotics_biodegradation_and_metabolism|L3__Aminobenzoate_degradation	3.1349	D	2.2736	0.0435
L1__Human_Diseases|L2__Immune_disease	0.0758	D	2.0908	0.0211
L1__Organismal_Systems|L2__Digestive_system|L3__Mineral_absorption	2.8283	D	2.0314	0.0355
L1__Cellular_Processes|L2__Transport_and_catabolism|L3__Endocytosis	0.5879	D	2.0025	0.0261
L1__Metabolism|L2__Biosynthesis_of_other_secondary_metabolites|L3__Streptomycin_biosynthesis	4.3150	H	3.3849	0.0337
L1__Metabolism|L2__Lipid_metabolism|L3__Fatty_acid_biosynthesis	4.3225	H	3.3541	0.0079
L1__Metabolism|L2__Glycan_biosynthesis_and_metabolism|L3__Lipopolysaccharide_biosynthesis	3.9296	H	3.2705	0.0165
L1__Metabolism|L2__Carbohydrate_metabolism|L3__Citrate_cycle_TCA_cycle	4.1255	H	3.1283	0.0109
L1__Metabolism|L2__Metabolism_of_cofactors_and_vitamins|L3__Vitamin_B6_metabolism	4.0404	H	2.9738	0.0163
L1__Metabolism|L2__Metabolism_of_cofactors_and_vitamins|L3__Folate_biosynthesis	3.9551	H	2.9312	0.0445
L1__Metabolism|L2__Metabolism_of_terpenoids_and_polyketides|L3__Zeatin_biosynthesis	3.7492	H	2.9154	0.0456
L1__Metabolism|L2__Energy_metabolism|L3__Carbon_fixation_in_photosynthetic_organisms	4.2837	H	2.8725	0.0149
L1__Metabolism|L2__Metabolism_of_terpenoids_and_polyketides|L3__Polyketide_sugar_unit_biosynthesis	3.6045	H	2.8196	0.0456
L1__Metabolism|L2__Glycan_biosynthesis_and_metabolism|L3__N_Glycan_biosynthesis	2.7062	H	2.2112	0.0148
L1__Metabolism|L2__Lipid_metabolism|L3__Steroid_hormone_biosynthesis	2.5557	H	2.1685	0.0119

Table S5 Enriched bacterial virulence factors in groups using LEfSe analysis

**Virulence Factor gene**	**description**	**keyword**	**max log mean**	**Group**	**Log (LDA score)**	**p-value (adjusted)**	**total**
spaG	SpaH-type pili minorsubunit SpaG, pilus tip protein	Adherence	2.59	A	2.06	0.01	Colonization, adherece and invasion (31)
p97	protein p97; cilium adhesin	Adherence	2.63	A	2.10	0.00	
lytC	1,4-beta-N-acetylmuramidase	Adherence	3.30	A	2.74	0.01	
pebA	bifunctional adhesin/ABC transporter aspartate/glutamate-binding protein	Adherence	3.73	A	2.87	0.04	
pilT	twitching motility protein PilT	Adherence	2.97	A	2.35	0.01	
htpB	Hsp60, 60K heat shock protein HtpB	Adherence	3.33	A	2.31	0.03	
cna	collagen adhesin precursor	Adherence	2.67	A	2.21	0.00	
papX	PapX protein regulates flagellum synthesis to repress motility	Adherence	2.96	A	2.43	0.04	
srtC	sortase	Adherence; Biofilm formation; Sortase-assembled pili	2.81	A	2.39	0.00	
ebpA	endocarditis and biofilm-associated pilus tip protein EbpA	Adherence; Biofilm formation; Sortase-assembled pili	2.55	A	2.05	0.00	
yhxB_manB	phosphomannomutase	Adherence; Endotoxin	3.36	A	2.48	0.01	
msbA	lipid transporter ATP-binding/permease	Adherence; Endotoxin	4.05	A	3.28	0.00	
orfM	deoxyribonucleotide triphosphate pyrophosphatase	Adherence; Endotoxin	3.51	A	2.46	0.02	
fctB	minor pilin FctB	Adherence; Sortase-assembled pili	2.57	A	2.17	0.00	
srtG1	sortase, SrtB family	Adherence; Sortase-assembled pili	2.95	A	2.34	0.01	
sipA	signal peptidase I	Adherence; Sortase-assembled pili	3.43	A	2.88	0.00	
srtC_1_srtB	sortase	Adherence; Sortase-assembled pili	2.65	A	2.20	0.00	

tapB	type IV pilus assembly ATPase, PilB-like	Adherence; Type IV pili	2.33	A	2.02	0.02	
fsrA	response regulator	Quorum sensing system; Two-component system	3.15	A	2.66	0.01	
bfmR	biofilm-controlling response regulator	Regulation; Two-component system	4.26	A	3.43	0.01	
clpC	endopeptidase Clp ATP-binding chain C	Stress protein	3.70	A	2.83	0.00	
gtcA	wall teichoic acid glycosylation protein GtcA	Invasion	3.11	A	2.62	0.01	
iap_cwhA	P60 extracellular protein, invasion associated protein Iap	Invasion	4.23	A	3.86	0.00	
inlA	Internalin A	Invasion	2.92	A	2.27	0.03	
flhF	flagellar biosynthesis protein	Secretion system; Invasion; Motility	3.63	A	2.74	0.00	
fleN	flagellar synthesis regulator FleN	Secretion system; Invasion; Motility	2.98	A	2.27	0.01	
virD4	type IV secretion system component VirD4	Secretion system; Type IV secretion system; Type IVa	4.04	A	3.41	0.04	
srtD	fimbrial associated sortase	Adherence	2.82	A	2.26	0.01	
essC	type VII secretion system protein EssC, FtsK/SpoIIIE family ATPase	Secretion system; Type VII secretion system	3.43	A	2.64	0.00	
esxA	type VII secretion system protein secreted protein EsxA	Secretion system; Type VII secretion system	2.51	A	2.08	0.00	
fss1	Enterococcus faecalis surface protein Fss1, fibrinogen binding protein	Adherence	3.26	A	2.67	0.04	
eccA1	ESX-1 type VII secretion system AAA+ ATPase EccA1	Secretion system; Type VII secretion system	3.21	A	2.73	0.00	Anti-phagocytosis (28)
yscN	type III secretion system ATPase YscN	Secretion system; Type III secretion system	3.14	A	2.58	0.01	
cdsD	Type III secretion system inner membrane ring complex protein CdsD	Secretion system; Type III secretion system	2.85	A	2.37	0.01	
pkn5	serine/threonine protein kinase	Secretion system; Type III secretion system	2.71	A	2.25	0.01	
cdsN	Type III secretion system ATPase	Secretion system; Type III	3.15	A	2.62	0.00	

		secretion system					
spa47	type III secretion system ATPase spa47	Secretion system; Type III secretion system	2.84	A	2.39	0.02	
coxH2	Coxiella Dot/Icm type IVB secretion system translocated effector	Secretion system; Type IV secretion system	3.49	A	2.65	0.01	
lirB	Dot/Icm type IV secretion system effector LirB	Secretion system; Type IV secretion system	3.68	A	2.68	0.03	
lpg2936	Dot/Icm type IV secretion system effector	Secretion system; Type IV secretion system	3.35	A	2.37	0.02	
wcbQ	capsular polysaccharide biosynthesis transmembrane protein	Antiphagocytosis	3.11	A	2.56	0.00	
wcbJ	capsular polysaccharide biosynthesis protein	Antiphagocytosis	2.71	A	2.20	0.04	
hasC	UDP-glucose pyrophosphorylase	Antiphagocytosis; Adherence; Tissue invasion	3.43	A	2.43	0.03	
glf	UDP-galactopyranose mutase	Antiphagocytosis; Serum resistance	3.19	A	2.51	0.01	
Cj1436c	aminotransferase	Antiphagocytosis; Serum resistance	3.53	A	2.61	0.04	
capA	CapA, required for Poly-gamma-glutamate transport	Antiphagocytosis; Serum resistance	3.18	A	2.37	0.04	
cps4A	capsular polysaccharide biosynthesis protein Cps4A	Antiphagocytosis; Serum resistance	3.15	A	2.57	0.00	
cps4E	capsular polysaccharide biosynthesis protein Cps4E	Antiphagocytosis; Serum resistance	2.79	A	2.27	0.04	
cps4H	capsular polysaccharide biosynthesis protein Cps4H	Antiphagocytosis; Serum resistance	3.22	A	2.50	0.01	
ctrD	capsule polysaccharide export ATP-binding protein CtrD	Antiphagocytosis; Serum resistance	2.56	A	2.07	0.00	
Cj1432c	sugar transferase	Antiphagocytosis; Serum resistance	2.63	A	2.18	0.03	
cap8B	capsular polysaccharide synthesis enzyme Cap8B	Antiphagocytosis; Serum	3.02	A	2.40	0.02	

		resistance					
cps4C	capsular polysaccharide biosynthesis protein Cps4C	Antiphagocytosis; Serum resistance	2.69	A	2.09	0.04	
cpsE	glycosyl transferase, group 2 family protein	Antiphagocytosis; Serum resistance	3.34	A	2.69	0.00	
cpsG	MurB family protein	Antiphagocytosis; Serum resistance	3.51	A	2.75	0.00	
cpsA	CpsA responsible for transfer of CPS from the membrane lipid to the cell wall peptidoglycan	Antiphagocytosis; Serum resistance	3.49	A	2.56	0.01	
cpsC	teichoic acid biosynthesis protein, putative	Antiphagocytosis; Serum resistance	2.99	A	2.47	0.02	
lspA	signal peptidase II	Peptidase	3.35	A	2.60	0.00	
recN	DNA repair protein RecN	Stress protein	3.29	A	2.26	0.05	
fbpC	iron(III) ABC transporter, ATP-binding protein	Iron uptake; ABC transporter	4.58	A	3.68	0.01	Nutrition uptake, growth and spread (17)
hitC	iron(III) ABC transporter, ATP-binding protein	Iron uptake; ABC transporter	4.47	A	3.50	0.02	
feoB	ferrous iron transporter B	Iron uptake; Ferrous iron uptake	3.59	A	2.57	0.05	
feoA	ferrous iron transporter A	Iron uptake; Ferrous iron uptake	3.82	A	3.15	0.00	
hgpC	hemoglobin-binding protein	Iron uptake; Heme uptake	2.67	A	2.01	0.03	
srtB	NPQTN specific sortase B	Iron uptake; Heme uptake	3.11	A	2.54	0.02	
pchG	pyochelin biosynthetic protein PchG	Iron uptake; Siderophore	1.17	A	2.02	0.04	
ybtP	lipoprotein inner membrane ABC-transporter	Iron uptake; Siderophore	3.48	A	2.83	0.01	
bauE	ferric siderophore ABC transporter, ATP-binding protein BauE	Iron uptake; Siderophore	3.78	A	2.54	0.05	
barA	siderophore efflux system of the ABC superfamily	Iron uptake; Siderophore	2.81	A	2.33	0.00	
barB	siderophore efflux system of the ABC superfamily	Iron uptake; Siderophore	2.47	A	2.02	0.05	
ideR	Iron-dependent repressor and activator IdeR	Regulation	3.60	A	3.06	0.01	
phoP	Possible two component system response transcriptional positive	Regulation	4.21	A	3.12	0.03	

	regulator PhoP						
allB	allantoinase	Nutritional factor	3.51	A	2.53	0.01	
ppkA	serine/threonine protein kinase PpkA	Secretion system; Type VI secretion system	3.69	A	3.12	0.01	
pppA	Pseudomonas protein phosphatase PppA	Secretion system; Type VI secretion system	3.42	A	2.88	0.00	
nagK	hyaluronidase	Exoenzyme; Carbohydrate-active enzyme; Hyaluronidase	2.82	A	2.31	0.01	
wzt	lipopolysaccharide O-antigen ABC transport system ATP-binding component	Endotoxin	3.32	A	2.67	0.01	Toxin and endotoxin (15)
wzm	lipopolysaccharide O-antigen ABC transport system transmembrane component	Endotoxin	3.09	A	2.51	0.00	
lpxE	phosphatidylglycerophosphatase B	Endotoxin	2.95	A	2.32	0.03	
wbbM	glycosyltransferase	Endotoxin	3.00	A	2.33	0.02	
fabZ	(3R)-hydroxymyristoyl ACP dehydratase	Endotoxin	3.41	A	2.69	0.03	
cyaB	cyclolysin secretion ATP-binding protein	Toxin; Adenylate cyclase; RTX toxin	3.45	A	2.65	0.00	
clbJ	colibactin non-ribosomal peptide synthetase ClbJ	Toxin; Genotoxin	2.61	A	2.10	0.00	
clbI	colibactin polyketide synthase ClbI	Toxin; Genotoxin	2.61	A	2.23	0.00	
clbM	precolibactin export MATE transporter ClbM	Toxin; Genotoxin	4.34	A	3.56	0.04	
clbL	colibactin biosynthesis amidase ClbL	Toxin; Genotoxin	3.14	A	2.52	0.00	
clbS	colibactin self-protection protein ClbS	Toxin; Genotoxin	2.80	A	2.26	0.00	
cylR2	cytolysin regulator R2	Toxin; Hemolysin/bacteriocin; Biofilm formation	3.91	A	3.23	0.01	
toxA	toxin A	Toxin; Intracellular toxin; Glucosyltransferase	2.98	A	2.50	0.00	
rtxB	RTX toxin transporter RtxB	Toxin; RTX toxin; Actin monomer cross-linking	3.26	A	2.54	0.02	

		activity					
rtxA	RTX toxin RtxA	Toxin; RTX toxin; Actin monomer cross-linking activity	2.58	A	2.18	0.01	
mucD	serine protease MucD precursor	None	3.55	A	2.75	0.01	Others (3)
mbtJ	Putative acetyl hydrolase MbtJ	None	2.82	A	2.18	0.03	
CBU_1434	Coxiella Dot/Icm type IVB secretion system translocated effector	Secretion system; Type IVBsecretion system	3.35	A	2.55	0.04	
IlpA	immunogenic lipoprotein A	Adherence	3.37	C	2.60	0.03	Colonization, adherece and invasion (73)
focA	F1C major fimbrial subunit precursor	Adherence	2.47	C	2.06	0.01	
lap	Listeria adhesion protein Lap	Adherence	4.01	C	3.20	0.00	
pebA	bifunctional adhesin/ABC transporter aspartate/glutamate-binding protein	Adherence	3.83	C	3.20	0.00	
cna	collagen adhesin precursor	Adherence	3.04	C	2.75	0.00	
papX	PapX protein regulates flagellum synthesis to repress motility	Adherence	2.96	C	2.38	0.00	
pilC	still frameshift type 4 fimbrial biogenesis protein PilC	Adherence	2.80	C	2.10	0.03	
pilD	type IV pilus prepilin peptidase	Adherence	2.78	C	2.27	0.05	
lmb	laminin-binding surface protein	Adherence	2.75	C	2.02	0.01	
cbpD	choline binding protein D	Adherence	2.67	C	2.15	0.01	
papC	usher protein PapC	Adherence	2.61	C	2.17	0.01	
pilV	type IV pilus biogenesis protein PilV	Adherence	2.49	C	2.11	0.02	
acfD	accessory colonization factor AcfD	Adherence	2.46	C	2.13	0.00	
spaG	SpaH-type pili minorsubunit SpaG, pilus tip protein	Adherence	2.68	C	2.29	0.00	
yapC	autotransporter protein YapC	Adherence; Autotransporter	3.11	C	2.66	0.01	
badA	Surface protein/Bartonella adhesin BadA	Adherence; Autotransporter	2.84	C	2.36	0.02	
icaB	N-deacetylase, involved in polysaccharide intercellular adhesin(PIA) synthesis	Adherence; Biofilm formation	2.86	C	2.37	0.00	
esp	Enterococcal surface protein; Esp	Adherence; Biofilm formation	2.48	C	2.14	0.02	
msbA	lipid transporter ATP-binding/permease	Adherence; Endotoxin	3.97	C	3.08	0.02	

rfaE	ADP-heptose synthase	Adherence; Endotoxin	3.39	C	2.66	0.00	
fimD	usher protein FimD	Adherence; Fimbrial	3.08	C	2.47	0.05	
lpfA	long polar fimbria protein LpfA	Adherence; Fimbrial	2.78	C	2.42	0.00	
csgC	curli assembly protein CsgC	Adherence; Fimbrial	2.57	C	2.12	0.01	
csgF	curli production assembly/transport protein CsgF	Adherence; Fimbrial	2.47	C	2.03	0.01	
oapA	opacity associated protein (oapA)	Adherence; Nonfimbrial structure	2.81	C	2.23	0.04	
shdA	AIDA autotransporter-like protein	Adherence; Nonfimbrial; Autotransporter	3.05	C	2.65	0.02	
misL	putative autotransporter	Adherence; Nonfimbrial; Autotransporter	2.59	C	2.17	0.01	
ompU	outer membrane protein OmpU	Adherence; Porin; Immunostimulatory molecule	3.15	C	2.58	0.03	
pilB	major subunit PilB	Adherence; Sortase-assembled pili	2.92	C	2.46	0.00	
tapD	prepilin peptidase, pilD-like	Adherence; Type IV pili	3.07	C	2.42	0.04	
tapT	twitching ATPase	Adherence; Type IV pili	2.80	C	2.27	0.01	
tapM	type IV pilus assembly protein PilM	Adherence; Type IV pili	2.65	C	2.23	0.00	
tapQ	type IV pilus secretin PilQ	Adherence; Type IV pili	2.63	C	2.06	0.05	
fliQ	flagellar biosynthetic protein FliQ	Secretion system; Invasion; Motility	3.18	C	2.71	0.01	
flgM	negative regulator of flagellin synthesis	Secretion system; Invasion; Motility	3.07	C	2.65	0.01	
fliE	flagellar hook-basal body complex protein FliE	Secretion system; Invasion; Motility	3.01	C	2.43	0.02	
flgC	flagellar basal-body rod protein FlgC	Secretion system; Invasion; Motility	3.00	C	2.49	0.01	
flgB	flagellar basal body rod protein FlgB	Secretion system; Invasion; Motility	2.94	C	2.37	0.01	

cheB	chemotaxis-specific methylesterase	Secretion system; Invasion; Motility	2.92	C	2.37	0.00	
fliN	flagellar motor switch protein FliN	Secretion system; Invasion; Motility	2.89	C	2.38	0.00	
flgG	flagellar basal body rod protein FlgG	Secretion system; Invasion; Motility	2.80	C	2.17	0.03	
fliS	flagellar protein FliS	Secretion system; Invasion; Motility	2.75	C	2.16	0.04	
flhE	flagellar biosynthesis protein FlhE	Secretion system; Invasion; Motility	2.72	C	2.25	0.01	
fliT	flagellar protein FliT	Secretion system; Invasion; Motility	2.71	C	2.25	0.00	
fliF	flagellar M-ring protein FliF	Secretion system; Invasion; Motility	2.69	C	2.18	0.00	
fliP	flagellar biosynthesis protein FliP	Secretion system; Invasion; Motility	2.69	C	2.18	0.01	
fliJ	flagellar protein FliJ	Secretion system; Invasion; Motility	2.67	C	2.23	0.01	
fliL	flagellar basal body protein FliL	Secretion system; Invasion; Motility	2.66	C	2.21	0.01	
flhD	flagellar transcriptional activator FlhD	Secretion system; Invasion; Motility	2.62	C	2.16	0.01	
flgA	flagellar basal body P-ring biosynthesis protein FlgA	Secretion system; Invasion; Motility	2.61	C	2.23	0.00	
fliM	flagellar motor switch protein FliM	Secretion system; Invasion; Motility	2.60	C	2.03	0.01	
flgI	flagellar P-ring protein precursor FlgI	Secretion system; Invasion; Motility	2.60	C	2.18	0.00	
flgF	flagellar basal-body rod protein	Secretion system; Invasion; Motility	2.59	C	2.14	0.00	

flgN	flagella synthesis protein FlgN	Secretion system; Invasion; Motility	2.57	C	2.12	0.01	
flgE	flagellar hook protein FlgE	Secretion system; Invasion; Motility	2.57	C	2.06	0.00	
fleN	flagellar synthesis regulator FleN	Motility	2.98	C	2.32	0.00	
fliI	flagellum-specific ATP synthase FliI	Motility	2.95	C	2.37	0.00	
fliO	flagellar biosynthesis protein, FliO	Motility	2.90	C	2.45	0.00	
flhB	flagellar protein FlhB	Motility	2.80	C	2.24	0.01	
flgD	flagellar hook capping protein	Motility	2.75	C	2.21	0.01	
bcfD	fimbrial adhesin protein	Motility	2.55	C	2.16	0.00	
bcfA	fimbrial subunit	Motility	2.47	C	2.04	0.00	
bcfE	fimbrial subunit	Motility	2.68	C	2.16	0.04	
lafK	two-component system lateral flagellar response regulator	Motility; Colonization	2.87	C	2.37	0.01	
lfhA	lateral flagellar biosynthesis protein	Motility; Colonization	2.65	C	2.21	0.01	
lafU	LafU, chemotaxis protein	Motility; Colonization	2.51	C	2.07	0.00	
lfiE	lateral flagellar hook basal body protein, LfiE	Motility; Colonization	2.45	C	2.12	0.02	
fliG	flagellar motor switch protein G	Motility	2.72	C	2.20	0.01	
rck	resistance to complement killing	Serum resistance; Adherence; Invasion	3.03	C	2.68	0.00	
lpeA	lipoprotein promoting cell invasion	Invasion	2.80	C	2.22	0.03	
mrkJ	phosphodiesterase	Biofilm formation	2.64	C	2.07	0.05	
bopD	sugar-binding transcriptional regulator, LacI family	Biofilm formation	4.32	C	3.69	0.00	
ureG	urease accessory protein (ureG)	Enzyme; Acid resistance; Colonization	3.11	C	2.49	0.01	
coxH2	Coxiella Dot/Icm type IVB secretion system translocated effector	Secretion system; Type IV secretion system	3.50	C	2.72	0.01	Anti-phagocytosis (18)
lpg0257	Dot/Icm type IV secretion system effector	Secretion system; Type IV secretion system	3.25	C	2.74	0.02	
dotD	Dot/Icm type IV secretion system outer membrane subcomplex protein DotD	Secretion system; Type IV secretion system	2.75	C	2.28	0.04	

icmP_dotM	Dot/Icm type IV secretion system protein IcmP/DotM	Secretion system; Type IV secretion system	2.52	C	2.11	0.01	
cdsN	Type III secretion system ATPase	Secretion system; Type III secretion system	3.03	C	2.44	0.03	
cdsD	Type III secretion system inner membrane ring complex protein CdsD	Secretion system; Type III secretion system	2.71	C	2.27	0.02	
dotC	Type IVB secretion system protein DotC, outer membrane lipoprotein of core-transmembrane complex	Secretion system; Type IV secretion system; Type IVb	2.56	C	2.13	0.01	
dotB	Type IVB secretion system Dot/Icm ATPase DotB	Secretion system; Type IV secretion system; Type IVb	2.52	C	2.09	0.02	
AHA_1846	Type VI secretion system protein	Secretion system; Type VI secretion system	2.79	C	2.35	0.01	
vgrG	VgrG protein	Secretion system; Type VI secretion system	2.70	C	2.29	0.02	
AHA_1843	Type VI secretion system protein	Secretion system; Type VI secretion system	2.54	C	2.08	0.03	
eccCa1	ESX-1 type VII secretion system FtsK/SpoIIIE family protein EccCa1	Secretion system; Type VII secretion system	2.51	C	2.21	0.03	
wcbR	capsular polysaccharide biosynthesis fatty acid synthase	Antiphagocytosis	2.95	C	2.31	0.03	
hasC	UDP-glucose pyrophosphorylase	Antiphagocytosis; Adherence; Tissue invasion	3.43	C	2.52	0.01	
gnd	6-phosphogluconate dehydrogenase	Antiphagocytosis; Serum resistance	3.35	C	2.66	0.00	
cps4A	capsular polysaccharide biosynthesis protein Cps4A	Antiphagocytosis; Serum resistance	2.99	C	2.30	0.04	
wcaG	GDP-fucose synthetase	Antiphagocytosis; Serum resistance	2.55	C	2.09	0.01	
manB	phosphomannomutase	None	3.02	C	2.43	0.00	
entB	2,3-dihydro-2,3-dihydroxybenzoate synthetase, isochroismatase	Iron acquision; Siderophore	3.23	C	2.71	0.02	Nutrition uptake,
fepD	iron-enterobactin transporter membrane protein	Iron acquision; Siderophore	3.15	C	2.62	0.01	

fepG	iron-enterobactin transporter permease	Iron acquision; Siderophore	2.80	C	2.29	0.02	growth and spread (24)
fbpC	iron(III) ABC transporter, ATP-binding protein	Iron uptake; ABC transporter	4.62	C	3.86	0.00	
hitC	iron(III) ABC transporter, ATP-binding protein	Iron uptake; ABC transporter	4.48	C	3.57	0.01	
shuV	ATP-binding component of hemin transport system	Iron uptake; Heme uptake	3.25	C	2.53	0.03	
chuT	periplasmic heme-binding protein ChuT	Iron uptake; Heme uptake	2.93	C	2.28	0.03	
entE	non-ribosomal peptide synthetase adenylate-forming enzyme of acinetobactin synthesis	Iron uptake; Siderophore	3.36	C	2.52	0.03	
entD	phosphopantetheinyl transferase component of enterobactin synthase multienzyme complex	Iron uptake; Siderophore	2.91	C	2.35	0.05	
entC	isochorismate synthase 1	Iron uptake; Siderophore	2.77	C	2.27	0.01	
barB	siderophore efflux system of the ABC superfamily	Iron uptake; Siderophore	2.49	C	2.07	0.00	
ybtS	salicylate synthase Irp9	Iron uptake; Siderophore	3.05	C	2.15	0.03	
lipF	Probable esterase/lipase LipF	Metabolic adaptation	2.94	C	2.28	0.03	
allR	DNA-binding transcriptional repressor AllR	Nutritional factor	3.57	C	2.98	0.01	
allD	ureidoglycolate dehydrogenase	Nutritional factor	3.22	C	2.59	0.02	
allA	ureidoglycolate hydrolase	Nutritional factor	2.60	C	2.06	0.03	
msrA_B_pilB	trifunctional thioredoxin/methionine sulfoxide reductase A/B protein	Stress protein	3.45	C	2.70	0.03	
lplA1	lipoate protein ligase	Intracellular growth	3.05	C	2.30	0.01	
irtB	Iron-regulated transporter IrtB	Iron-regulated	2.92	C	2.37	0.00	
entS	enterobactin exporter, iron-regulated	Iron-regulated	2.66	C	2.23	0.01	
icl	Isocitrate lyase Icl (isocitrase) (isocitratase)	Metabolic adaptation	3.04	C	2.52	0.01	
cheD	methyl-accepting chemotaxis protein CheD	chemotaxis	3.41	C	2.84	0.01	
cheR	chemotaxis methyltransferase CheR	chemotaxis	2.66	C	2.14	0.00	
cheZ	chemotaxis regulator CheZ	chemotaxis	2.50	C	2.05	0.00	
fabZ	(3R)-hydroxymyristoyl ACP dehydratase	Endotoxin	3.28	C	2.34	0.05	Toxin and endotoxin (12)
wbpL	undecaprenyl-phosphate alpha-N-acetylglucosaminyltransferase	Endotoxin	2.99	C	2.29	0.00	
bplB	probable acetyltransferase	Endotoxin	2.96	C	2.38	0.03	
pgm	phosphoglucomutase	Endotoxin	2.82	C	2.36	0.01	

wzz	positive regulator for alginate biosynthesis MucC	Endotoxin	2.80	C	2.34	0.01	
wbpB	lipopolysaccharide biosynthesis protein	Endotoxin	2.66	C	2.02	0.01	
cyaB	cyclolysin secretion ATP-binding protein	Toxin; Adenylate cyclase; RTX toxin	3.41	C	2.54	0.01	
clbB	colibactin hybrid non-ribosomal peptide synthetase/type I polyketide synthase ClbB	Toxin; Genotoxin	3.50	C	2.86	0.02	
clbD	colibactin biosynthesis dehydrogenase ClbD	Toxin; Genotoxin	3.29	C	2.63	0.01	
clbL	colibactin biosynthesis amidase ClbL	Toxin; Genotoxin	3.16	C	2.66	0.01	
clbA	colibactin biosynthesis phosphopantetheinyl transferase ClbA	Toxin; Genotoxin	3.02	C	2.48	0.00	
toxA	toxin A	Toxin; Intracellular toxin; Glucosyltransferase	2.79	C	2.25	0.03	
gspE	general secretion pathway protein E	Secretion system; Type II secretion system	2.81	C	2.18	0.03	Others (13)
gspK	general secretion pathway protein K	Secretion system; Type II secretion system	2.60	C	2.20	0.04	
gspL	general secretion pathway protein L	Secretion system; Type II secretion system	2.45	C	2.03	0.02	
espX4	Type III secretion system effector EspX4	Type III secretion system	2.53	C	2.11	0.04	
pilQ	type IV pilus secretin protein PilQ	Type IV secretion system	2.49	C	2.05	0.02	
etgA	T3SS-associated peptidoglycan lytic enzyme	Type III secretion system	2.77	C	2.30	0.01	
pilM	type IV pilus inner membrane platform protein PilM	Type IV secretion system	2.69	C	2.25	0.00	
pvdH	diaminobutyrate-2-oxoglutarate aminotransferase PvdH	None	3.78	C	2.81	0.04	
mucD	serine protease MucD precursor	None	3.52	C	2.63	0.01	
fleQ_flrC	FleQ protein	None	3.04	C	2.31	0.04	
phzF1	phenazine biosynthesis protein PhzF, isomerase	None	2.76	C	2.17	0.02	
mbtJ	Putative acetyl hydrolase MbtJ	None	2.68	C	2.01	0.04	
icsA_virG	autotransporter, actin tail assembly protein IcsA/VirG	None	2.52	C	2.13	0.01	
lap	Listeria adhesion protein Lap	Adherence	3.97	D	3.03	0.02	Colonization, adherece and invasion (14)
lmb	laminin-binding surface protein	Adherence	2.78	D	2.05	0.01	
p97	protein p97; cilium adhesin	Adherence	2.62	D	2.16	0.04	

pilT	twitching motility protein PilT	Adherence	2.81	D	2.08	0.01	
pce	choline binding protein E	Adherence	3.04	D	2.37	0.03	
msbA	lipid transporter ATP-binding/permease	Adherence; Endotoxin	3.97	D	3.09	0.01	
sipA	signal peptidase I	Adherence; Sortase-assembled pili	3.30	D	2.67	0.02	
gtcA	wall teichoic acid glycosylation protein GtcA	Invasion	2.99	D	2.42	0.03	
flhF	flagellar biosynthesis protein	Secretion system; Invasion; Motility	3.57	D	2.48	0.03	
fleN	flagellar synthesis regulator FleN	Secretion system; Invasion; Motility	2.97	D	2.29	0.00	
fliO	flagellar biosynthesis protein, FliO	Secretion system; Invasion; Motility	2.68	D	2.11	0.03	
fliI	flagellum-specific ATP synthase FliI	Secretion system; Invasion; Motility	2.91	D	2.23	0.01	
cheB	chemotaxis-specific methylesterase	Secretion system; Invasion; Motility	2.81	D	2.13	0.01	
fliB	flagellin lysine-N-methylase	Secretion system; Invasion; Motility	2.80	D	2.05	0.05	
wcbQ	capsular polysaccharide biosynthesis transmembrane protein	Antiphagocytosis	3.00	D	2.41	0.01	Anti-phagocytosis (13)
hasC	UDP-glucose pyrophosphorylase	Antiphagocytosis; Adherence; Tissue invasion	3.45	D	2.51	0.01	
cps4A	capsular polysaccharide biosynthesis protein Cps4A	Antiphagocytosis; Serum resistance	3.07	D	2.50	0.00	
cps4B	capsular polysaccharide biosynthesis protein Cps4B	Antiphagocytosis; Serum resistance	2.72	D	2.14	0.01	
cpsC	teichoic acid biosynthesis protein, putative	Antiphagocytosis; Serum resistance	2.81	D	2.16	0.02	
yscN	type III secretion system ATPase YscN	Secretion system; Type III secretion system	3.01	D	2.32	0.04	
cdsD	Type III secretion system inner membrane ring complex protein CdsD	Secretion system; Type III	2.64	D	2.15	0.01	

		secretion system					
cdsN	Type III secretion system ATPase	Secretion system; Type III secretion system	3.06	D	2.51	0.00	
coxH2	Coxiella Dot/Icm type IVB secretion system translocated effector	Secretion system; Type IV secretion system	3.46	D	2.48	0.02	
manB	phosphomannomutase	None	2.93	D	2.20	0.04	
galE	UDP-glucose 4-epimerase	None	3.37	D	2.40	0.05	
lpg2936	Dot/Icm type IV secretion system effector	Secretion system; Type IV secretion system	3.34	D	2.37	0.03	
lspA	signal peptidase II	Peptidase	3.32	D	2.56	0.00	
irtB	Iron-regulated transporter IrtB	Iron regulation	2.87	D	2.25	0.00	Nutrition uptake, growth and spread (13)
ideR	Iron-dependent repressor and activator IdeR	Iron regulation	3.46	D	2.75	0.01	
fbpC	iron(III) ABC transporter, ATP-binding protein	Iron uptake; ABC transporter	4.56	D	3.61	0.01	
hitC	iron(III) ABC transporter, ATP-binding protein	Iron uptake; ABC transporter	4.45	D	3.41	0.04	
feoA	ferrous iron transporter A	Iron uptake; Ferrous iron uptake	3.70	D	2.78	0.04	
srtB	NPQTN specific sortase B	Iron uptake; Heme uptake	3.00	D	2.40	0.04	
chuW	Putative oxygen independent coproporphyrinogen III oxidase	Iron uptake; Heme uptake	3.56	D	2.49	0.02	
barA	siderophore efflux system of the ABC superfamily	Iron uptake; Siderophore	2.64	D	2.04	0.01	
mgtB	Mg2+ transport protein	Magnesium uptake	4.00	D	2.86	0.02	
lipF	Probable esterase/lipase LipF	Metabolic adaptation	2.94	D	2.18	0.02	
ppkA	serine/threonine protein kinase PpkA	Secretion system; Type VI secretion system	3.49	D	2.78	0.03	
pppA	Pseudomonas protein phosphatase PppA	Secretion system; Type VI secretion system	3.26	D	2.62	0.01	
allB	allantoinase	Nutritional factor	3.52	D	2.61	0.01	
eccA1	ESX-1 type VII secretion system AAA+ ATPase EccA1	Secretion system; Type VII secretion system	3.13	D	2.62	0.03	Toxin/ endotoxin/

essC	type VII secretion system protein EssC, FtsK/SpoIIIE family ATPase	Secretion system; Type VII secretion system	3.39	D	2.59	0.00	(9)
bplB	probable acetyltransferase	Endotoxin	2.91	D	2.19	0.02	
lpxE	phosphatidylglycerophosphatase B	Endotoxin	2.91	D	2.25	0.02	
fabZ	(3R)-hydroxymyristoyl ACP dehydratase	Endotoxin	3.37	D	2.60	0.01	
cyaB	cyclolysin secretion ATP-binding protein	Toxin; Adenylate cyclase; RTX toxin	3.41	D	2.54	0.00	
clbA	colibactin biosynthesis phosphopantetheinyl transferase ClbA	Toxin; Genotoxin	2.86	D	2.08	0.04	
clbL	colibactin biosynthesis amidase ClbL	Toxin; Genotoxin	3.02	D	2.32	0.00	
toxA	toxin A	Toxin; Intracellular toxin; Glucosyltransferase	2.83	D	2.32	0.00	
mbtJ	Putative acetyl hydrolase MbtJ	None	2.73	D	2.12	0.01	Others (3)
mucD	serine protease MucD precursor	None	3.49	D	2.51	0.00	
srtD	fimbrial associated sortase	None	2.72	D	2.19	0.04	

Table S6 Enriched CAZy families in groups using LEfSe analysis

Taxa	Abundace	Group	LDA_score	p value
L1__AA|L2__AA6	3.4147598	A	2.681804	0.017097
L1__CBM|L2__CBM14	3.5284175	A	2.661391	0.008266
L1__CBM|L2__CBM2	3.598018	A	2.779788	0.022486
L1__CBM|L2__CBM22	2.7715603	A	2.142679	0.022486
L1__CBM|L2__CBM48	3.773349	A	2.957323	0.004425
L1__CBM|L2__CBM50	4.7293339	A	3.976684	0.004425
L1__CBM|L2__CBM65	2.659728	A	2.188461	0.029295
L1__CBM|L2__CBM79	2.3574847	A	2.0065	0.005602
L1__CE|L2__CE4	3.9511105	A	2.81352	0.005192
L1__GH|L2__GH112	2.90678	A	2.483135	0.002279
L1__GH|L2__GH120	2.5406368	A	2.015571	0.007096
L1__GH|L2__GH17	3.3754136	A	2.711946	0.011134
L1__GH|L2__GH42	3.6692895	A	2.903192	0.014855
L1__GH|L2__GH45	2.8305806	A	2.357838	0.025696
L1__GH|L2__GH65	3.9179201	A	2.949775	0.008266
L1__GH|L2__GH71	3.2779808	A	2.561341	0.017097
L1__GH|L2__GH72	3.5436616	A	2.424103	0.025696
L1__GH|L2__GH75	2.7924613	A	2.403161	0.011079
L1__GH|L2__GH77	3.4046038	A	2.680436	0.003762
L1__GT|L2__GT28	4.0239066	A	2.838902	0.019631
L1__GT|L2__GT29	3.2766813	A	2.270991	0.004425
L1__GT|L2__GT34	3.0414208	A	2.353563	0.019631
L1__GT|L2__GT47	3.3301458	A	2.475269	0.001611
L1__GT|L2__GT5	4.0108868	A	3.153132	0.005192
L1__GT|L2__GT60	2.4978247	A	2.107092	0.006077
L1__GT|L2__GT66	3.602778	A	2.563	0.012876
L1__GT|L2__GT7	3.2163769	A	2.01935	0.048341
L1__GT|L2__GT80	3.258821	A	2.392674	0.007096
L1__GT|L2__GT96	3.0189927	A	2.280846	0.025696
L1__AA|L2__AA1	3.0202291	C	2.429022	0.01703
L1__AA|L2__AA2	2.5925808	C	2.024115	0.015611
L1__AA|L2__AA4	3.0293562	C	2.399438	0.002257
L1__CE|L2__CE16	2.9168885	C	2.368433	0.025947
L1__GH|L2__GH1	4.0983551	C	3.317108	0.003423
L1__GH|L2__GH103	3.075738	C	2.490823	0.009968
L1__GH|L2__GH111	2.5091502	C	2.109421	0.001466
L1__GH|L2__GH135	2.9596041	C	2.288439	0.045038
L1__GH|L2__GH32	4.2460266	C	3.275436	0.038647
L1__GH|L2__GH42	3.8699339	C	3.328509	0.000413
L1__GH|L2__GH47	3.1175657	C	2.357313	0.010923
L1__GH|L2__GH53	3.947162	C	3.079726	0.033044
L1__GH|L2__GH65	3.9192715	C	3.00999	0.001466

L1__GH|L2__GH71	3.1661979	C	2.204436	0.028151
L1__GH|L2__GH73	3.9774757	C	2.986905	0.028151
L1__GH|L2__GH84	3.3232936	C	2.352775	0.041739
L1__GH|L2__GH93	3.4774043	C	2.536555	0.045038
L1__GT|L2__GT20	3.355823	C	2.510907	0.021985
L1__GT|L2__GT22	3.3048956	C	2.720892	0.025947
L1__GT|L2__GT34	3.0367053	C	2.328916	0.001635
L1__GT|L2__GT39	2.8096733	C	2.183278	0.028151
L1__GT|L2__GT4	5.0332217	C	3.869176	0.00379
L1__GT|L2__GT5	3.9509454	C	2.776093	0.014298
L1__GT|L2__GT51	4.3274706	C	3.113618	0.009968
L1__GT|L2__GT57	3.2335348	C	2.426113	0.038647
L1__GT|L2__GT77	4.0819696	C	3.74364	0.001822
L1__GT|L2__GT87	3.525767	C	2.868887	0.025947
L1__GT|L2__GT96	3.0106193	C	2.264763	0.01196
L1__AA|L2__AA4	2.9106742	D	2.193128	0.040852
L1__AA|L2__AA6	3.3918557	D	2.631918	0.011284
L1__CBM|L2__CBM14	3.4736032	D	2.510871	0.019913
L1__CBM|L2__CBM48	3.7285164	D	2.817317	0.003504
L1__CBM|L2__CBM50	4.6020179	D	3.307748	0.006651
L1__GH|L2__GH1	4.0515769	D	3.228395	0.029732
L1__GH|L2__GH103	2.8909104	D	2.161683	0.027852
L1__GH|L2__GH111	2.4933755	D	2.060189	0.000273
L1__GH|L2__GH112	2.7621574	D	2.299969	0.001039
L1__GH|L2__GH13	4.4151854	D	3.26698	0.016166
L1__GH|L2__GH23	4.5252398	D	3.444768	0.038382
L1__GH|L2__GH42	3.6840028	D	2.969879	0.002507
L1__GH|L2__GH65	3.8919401	D	2.861082	0.018587
L1__GH|L2__GH71	3.2744236	D	2.600246	0.010482
L1__GH|L2__GH72	3.5333219	D	2.420744	0.046198
L1__GH|L2__GH73	4.0360205	D	3.216496	0.002302
L1__GH|L2__GH77	3.3113564	D	2.473398	0.043456
L1__GT|L2__GT29	3.2665653	D	2.2237	0.006152
L1__GT|L2__GT34	2.9957952	D	2.295566	0.009029
L1__GT|L2__GT4	5.0082065	D	3.725325	0.043456
L1__GT|L2__GT47	3.3002298	D	2.386333	0.013053
L1__GT|L2__GT5	3.9841039	D	3.04319	0.003504
L1__GT|L2__GT66	3.5715695	D	2.473304	0.040852
L1__GT|L2__GT80	3.2298032	D	2.272254	0.029732
L1__GT|L2__GT96	3.0327675	D	2.361024	0.006651
L1__CBM|L2__CBM12	3.1709363	H	2.515113	0.013153
L1__CBM|L2__CBM18	3.6513607	H	3.252412	0.000955
L1__CBM|L2__CBM20	3.5654186	H	2.799938	0.028395
L1__CBM|L2__CBM27	2.6127728	H	2.142823	0.000505

L1__CBM|L2__CBM30	1.0948004	H	2.149387	0.000234
L1__CBM|L2__CBM6	3.5817938	H	2.768732	0.001192
L1__CBM|L2__CBM66	2.6219406	H	2.089692	0.010991
L1__CBM|L2__CBM73	2.9459548	H	2.551962	0.004264
L1__CE|L2__CE15	2.720007	H	2.215194	0.018674
L1__CE|L2__CE3	2.9162844	H	2.349855	0.042943
L1__CE|L2__CE8	3.4508578	H	2.766252	0.005179
L1__GH|L2__GH105	3.708372	H	3.19842	0.006739
L1__GH|L2__GH106	2.965373	H	2.512933	0.007768
L1__GH|L2__GH115	3.0314945	H	2.556172	0.005722
L1__GH|L2__GH12	3.4195935	H	2.853934	0.007892
L1__GH|L2__GH123	2.815602	H	2.367925	0.028196
L1__GH|L2__GH20	3.7534189	H	3.173442	0.015759
L1__GH|L2__GH28	3.8982696	H	3.086083	0.001641
L1__GH|L2__GH29	3.6627967	H	3.167579	0.026124
L1__GH|L2__GH57	2.7103967	H	2.21255	0.037614
L1__GH|L2__GH66	3.0205814	H	2.469979	0.013588
L1__GH|L2__GH78	3.9375491	H	3.128304	0.009646
L1__GH|L2__GH92	4.004253	H	3.343209	0.027455
L1__GH|L2__GH95	3.5306761	H	2.968566	0.003415
L1__GH|L2__GH97	3.5985348	H	3.193414	0.001689
L1__GT|L2__GT23	2.7516696	H	2.389376	0.000918
L1__GT|L2__GT3	2.5417921	H	2.129148	0.001984
L1__GT|L2__GT30	3.6549635	H	2.817754	0.010201
L1__GT|L2__GT32	2.9566617	H	2.367441	0.017216
L1__GT|L2__GT83	3.5086342	H	2.741122	0.000882
L1__GT|L2__GT90	3.9214456	H	3.172276	0.00045
L1__PL|L2__PL10	3.0409283	H	2.648174	0.005659
L1__PL|L2__PL14	1.301114	H	2.158041	0.029723
